# Shared Treg dysregulation underlies psoriasis vulgaris and atopic dermatitis through the PHF19-PRC2 epigenetic axis

**DOI:** 10.3389/fimmu.2026.1932788

**Published:** 2026-08-20

**Authors:** Yiting Lin, Xiaolei Su, Mingzhu Jin, Yuqi Wang, Yaoyao Li, Bingquan Zong, Haoran Song, Yajie Lv, Zhenhua Liu, Gang Wang, Shengxi Wu

**Affiliations:** 1 Department of Neurobiology, School of Basic Medicine, Fourth Military Medical University, Xi’an, China; 2 Department of Dermatology, Tangdu Hospital, Fourth Military Medical University, Xi’an, Shaanxi, China; 3 Department of Dermatology, Xijing Hospital, Fourth Military Medical University, Xi’an, Shaanxi, China; 4 School of Basic Medicine, Fourth Military Medical University, Xi’an, Shaanxi, China

**Keywords:** atopic dermatitis, PHF19, PRC2, psoriasis vulgaris, regulatory T cell, resveratrol

## Abstract

**Background:**

Psoriasis vulgaris (PV) and atopic dermatitis (AD) are chronic inflammatory skin diseases characterized by distinct dominant T cell subsets, yet emerging evidence suggests overlapping immune mechanisms. Whether a shared Treg abnormality underlies both diseases remains unclear.

**Methods:**

Single-cell RNA sequencing data from lesional T cells of 8 PV, 7 AD, and 7 healthy controls were analyzed. Treg subset identification, trajectory, communication, and enrichment analyses were conducted. Differentially expressed genes from both diseases were intersected and subjected to Mendelian randomization to screen for shared causal genes, followed by interaction mapping, drug prediction, and molecular docking. Findings were validated in Foxp3-tdTomato reporter mouse models of imiquimod-induced psoriasis-like and MC903-induced AD-like dermatitis by flow cytometry and quantitative PCR.

**Results:**

A shared Treg triad remodeling pattern was identified: central memory Treg (cmTreg) was almost completely depleted, cycling Treg (Treg-c) expanded, and effector Treg (eTreg) accumulated in both diseases. Pseudotemporal analysis revealed a differentiation coordination defect, with upstream Treg-c shifting prematurely while effector Treg failed terminal maturation. Cell communication analysis identified enhanced MIF-(CD74+CXCR4/CD44) signaling as a shared intercellular remodeling axis, with the network shifting from a cmTreg-centric topology to one jointly centered on Treg-c and eTreg. Mendelian randomization identified seven shared causal genes (PDCD5, PHF19, GNAQ, LRR1, DHX36, TMEM107, FAM200B), with PHF19 serving as a core accessory subunit of PRC2. Molecular docking and *in vivo* experiments demonstrated that resveratrol binds PHF19, is predicted to activate the PHF19-PRC2-H3K27me3 axis, restores Treg subset homeostasis, and ameliorates skin lesions in both models.

**Conclusions:**

Treg subset remodeling is a shared pathogenic feature, nominating PHF19-targeted activation as a tolerance-restoring therapeutic strategy.

## Graphical Abstract

**Figure:**
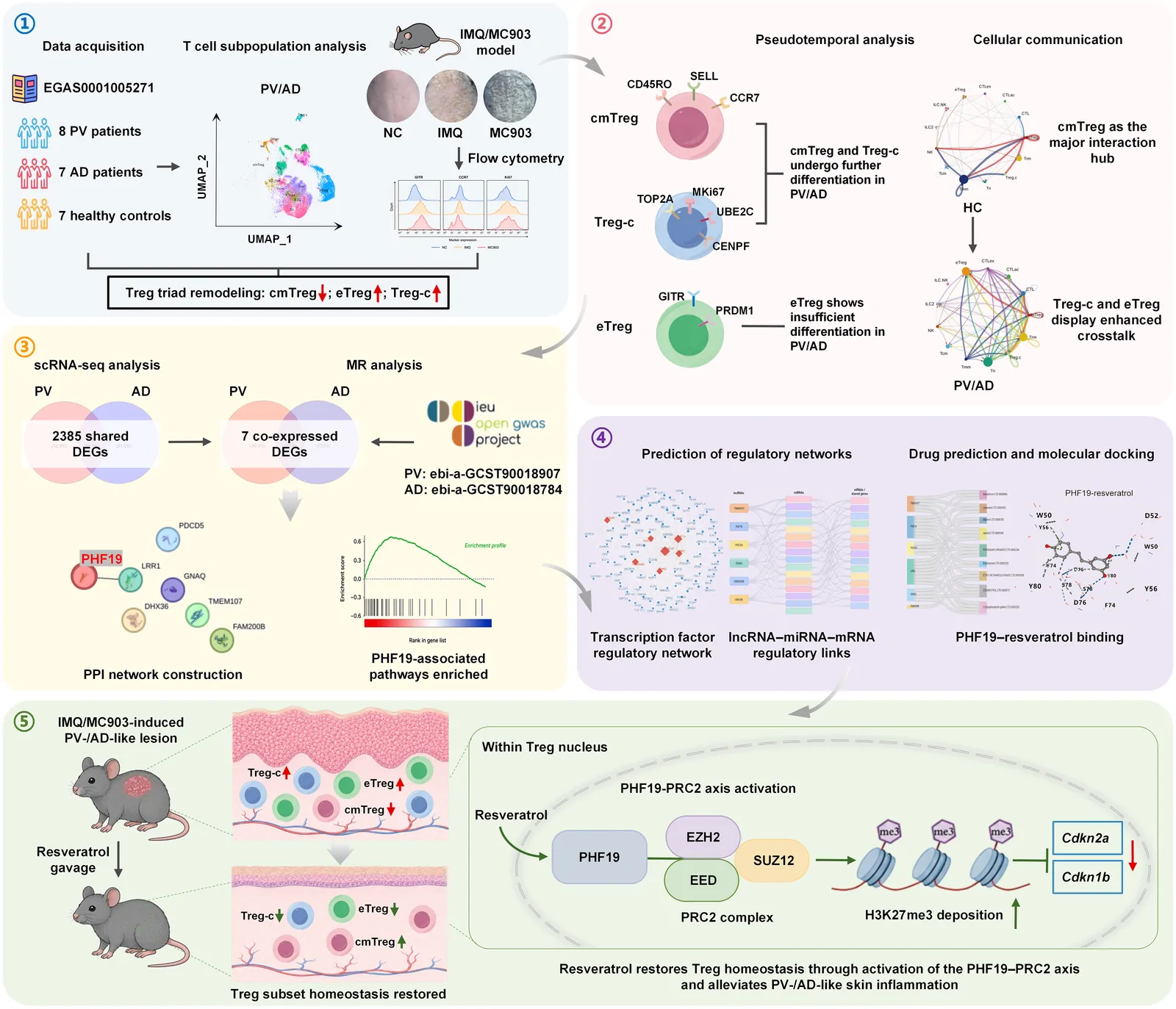
Integrative scRNA-seq and MR reveal shared Treg pathogenesis in psoriasis vulgaris and atopic dermatitis. scRNA-seq of lesional skin T cells identified a shared Treg triad remodeling pattern (cmTreg depletion, Treg-c expansion, eTreg accumulation) with defective differentiation trajectories and dysregulated cell-cell communication. MR analysis identified seven shared causal genes centered on PHF19, a core PRC2 accessory subunit. Molecular docking and *in vivo* validation in Foxp3-tdTomato mouse models demonstrated that resveratrol binds PHF19 with high affinity, activates the PHF19-PRC2-H3K27me3 axis, restores Treg subset homeostasis, and ameliorates skin lesions in both disease models.

## Introduction

Psoriasis vulgaris (PV) and atopic dermatitis (AD) are the most common chronic inflammatory skin diseases. Previously, these conditions were considered immunologically distinct: PV was thought to be driven primarily by T helper 1 (Th1) and T helper 17 (Th17) immune pathways, while AD was characterized by T helper 2 (Th2)-dominated immune responses (1, 2). However, mounting evidence has revealed substantial overlap between the two diseases across immune pathways, metabolic features, skin microbiome composition, and clinical manifestations (3, 4). Indeed, some researchers have proposed that PV and AD represent different presentations along a disease spectrum (5). Of particular note, biologic therapies targeting specific cytokines have further highlighted this intrinsic connection: anti-IL-17/IL-23 biologics used to treat PV can occasionally induce AD-like dermatitis (6, 7), while AD-targeted therapies such as dupilumab (anti-IL-4Rα) may induce psoriatic lesions (8, 9). This “immune drift” phenomenon suggests a deeper, inadequately understood interconnection between PV and AD at the level of immune homeostatic networks.

Regulatory T cells (Treg) serve as central guardians of immune tolerance (10, 11), and their functional impairment has been documented in both PV and AD. In psoriatic lesions, Tregs exhibit markedly attenuated capacity to suppress Th17 cells (12, 13), while in AD, Treg functional defects correlate closely with disease severity, leaving Th2-driven inflammation inadequately controlled (4, 14). We therefore hypothesize that this shared, intrinsic Treg dysfunction constitutes the cellular basis of immune drift: biologics potently suppress the targeted dominant pathway (e.g., Th17) yet fail to correct the underlying Treg defect, thereby unmasking a latent incapacity to restrain alternative inflammatory axes (e.g., Th2) (15). However, most prior Treg studies in PV and AD have relied on peripheral blood or bulk tissue analyses. While recent investigations have begun to elucidate the heterogeneity of skin-resident Tregs under homeostatic conditions, identifying tissue-adapted subsets with distinct transcriptional signatures (16, 17), the heterogeneity, differentiation trajectories, and functional states of Treg subsets within PV and AD lesional skin remain largely unexplored (4, 18).

Single-cell RNA sequencing (scRNA-seq) offers a powerful means to resolve Treg heterogeneity by profiling individual cells at unprecedented resolution within the lesional microenvironment (19). By identifying disease-specific Treg subsets and their key gene signatures, scRNA-seq enables precise characterization of functional states. However, correlation alone cannot establish causality (20, 21). Mendelian randomization (MR) analysis leverages genetic variants as instrumental variables to infer causal relationships between gene expression and disease, thereby providing mechanistic validation for candidate genes identified through scRNA-seq screening (22, 23).

Therefore, this study integrates scRNA-seq and MR analysis to systematically investigate shared Treg-related pathogenic mechanisms in PV and AD. We aim to (i) characterize Treg subset composition, differentiation trajectories, and functional states in lesional skin at single-cell resolution; (ii) identify shared co-expressed genes and establish their causal relevance through MR; (iii) elucidate the regulatory networks and candidate targeting drugs for these shared genes; and (iv) experimentally validate drug efficacy using established mouse models. Through these complementary approaches, this study not only provides novel insights into the shared pathogenesis of PV and AD but also offers potential therapeutic targets and drug candidates for restoring Treg function and fundamentally reestablishing immune homeostasis, addressing the immune drift challenges faced by current biologic therapies.

## Methods

### Animals and treatment

Foxp3-IREES-tdTomato reporter mice on the C57BL/6 background were purchased from the Shanghai Model Organisms Center, Inc. (Stock NM-KI-190119) and bred at the Animal Experiment Center of Air Force Medical University (License No. SYXK (Shaanxi)-2024-003). Female mice aged 6–8 weeks (18–20 g body weight) were used for all experiments. Mice were housed under specific-pathogen-free conditions with a 12-hour light/dark cycle at 22 ± 2 °C, with ad libitum access to standard chow and water. All animal experiments were performed in accordance with guidelines approved by the Animal Ethics Committee of the Fourth Military Medical University, Xi’an, China (Approval No. 20250234). Mice were randomly divided into five groups (n = 5 per group): (i) control (PBS), (ii) imiquimod (IMQ), (iii) MC903, (iv) IMQ plus resveratrol, and (v) MC903 plus resveratrol.

Resveratrol (MCE, Cat. No. HY-16561) was dissolved in dimethyl sulfoxide (DMSO; Sigma-Aldrich, Cat. No. D2650) and further diluted in PBS to a working concentration of 50 mg/kg. The dose selection was based on the following rationale: (i) Pharmacokinetic studies have shown that oral resveratrol exhibits very low bioavailability in humans due to extensive hepatic glucuronidation and sulfation (24). However, in murine models, higher doses (25–100 mg/kg) are routinely employed to achieve physiologically relevant plasma concentrations. We selected 50 mg/kg as a mid-range dose expected to produce plasma concentrations in the low micromolar range, consistent with concentrations demonstrated to modulate inflammatory pathways and chromatin-associated protein function *in vitro* (5–10 μM). This Cmax exceeds the concentrations required for *in vitro* modulation of chromatin readers and inflammatory mediators (5–10 μM). (ii) The study by Yao et al. (25) demonstrated that 50 mg/kg resveratrol significantly attenuates inflammatory pathology in a DSS-induced ulcerative colitis mouse model by restoring Treg/Th17 balance and suppressing colonic inflammatory cytokine production. This study provides direct precedent for the anti-inflammatory efficacy of 50 mg/kg resveratrol in a chronic inflammatory disease model, supporting its translational application to inflammatory skin disease. Three days before model establishment, mice in the intervention groups received resveratrol (200 μL, 50 mg/kg) daily by intragastric administration, whereas control mice received an equal volume of PBS. Dorsal hair was shaved 1–2 days prior to modeling. IMQ cream (62.5 mg; MedShine, Cat. No. S20040051) was applied topically to the shaved dorsal skin daily for 5 consecutive days to induce a psoriasis-like model (1). MC903 (calcipotriol; 200 μL; MCE, Cat. No. HY-10009) was applied daily for 15 consecutive days to induce an atopic dermatitis-like model (2). Resveratrol or PBS was administered concomitantly in intervention groups. After model construction, mice were anesthetized with tribromoethanol (Avertin; Sigma-Aldrich, Cat. No. M2960) and sacrificed by cervical dislocation. Dorsal skin tissues were collected for subsequent analyses.

### Histopathological analysis

Dorsal skin tissues were fixed in 4% paraformaldehyde (Servicebio, Cat. No. G1101) for 24 h, dehydrated through graded ethanol series, embedded in paraffin (Leica), and sectioned at 4 μm. Sections were stained with hematoxylin and eosin (H&E; Servicebio, Cat. No. G1005) following standard protocols. Epidermal thickness was quantified from at least three random fields per section using NDP.view2 software (Hamamatsu Photonics).

### Cell isolation and flow cytometry

Dorsal skin tissues were minced and digested in Dulbecco’s Modified Eagle Medium (DMEM; Gibco, Cat. No. 11965092) containing 1 mg/mL collagenase type IV (Roche, Cat. No. 11249002001) and 100 ng/mL DNase I (Roche, Cat. No. 10104159001) at 37 °C for 2 h with shaking. Digestion was terminated by adding DMEM supplemented with 10% fetal bovine serum (FBS; Gibco, Cat. No. 10099141C). Single-cell suspensions were filtered through a 70 μm cell strainer (Corning, Cat. No. 431751), centrifuged at 1,400 rpm for 10 min, washed twice with PBS, and resuspended in PBS for staining.

For surface staining, cells were incubated with the following antibodies at room temperature for 30 min in the dark: anti-CD3 (clone 17A2, BioLegend, Cat. No. 100306), anti-CD4 (clone GK1.5, BioLegend, Cat. No. 100559), anti-CD8 (clone 53-6.7, BioLegend, Cat. No. 100744), anti-CCR7 (clone 4B12, BioLegend, Cat. No. 120119), anti-GITR (clone DTA-1, BioLegend, Cat. No. 126311), and 7AAD viability dye (BioLegend, Cat. No. 420404). After washing with PBS, cells were fixed and permeabilized using the eBioscience™ Foxp3/Transcription Factor Staining Buffer Set (Invitrogen, Cat. No. 00-5523-00) according to the manufacturer’s instructions. Intracellular staining was performed with anti-Ki-67 (clone 16A8, BioLegend, Cat. No. 652406) at room temperature for 30 min. Cells were washed, resuspended in PBS, and analyzed on a BD FACSCanto II flow cytometer (BD Biosciences). Data were analyzed using FlowJo software (v10.8.1; BD Biosciences).

### RNA isolation and quantitative real-time PCR

Spleens were harvested, minced, and gently homogenized to obtain splenocytes, which were subsequently passed through a 70 μm cell strainer to remove tissue debris and obtain a single-cell suspension. Red blood cells were lysed using ACK Lysing Buffer (Thermo Fisher Scientific, Cat. #A1049201), and the remaining cells were washed with phosphate-buffered saline. After counting, cells were stained with anti-CD4 antibody (clone GK1.5, BioLegend, Cat. No. 100438) at 4 °C for 30 minutes, followed by 7-AAD viability dye (BioLegend, Cat. No. 420404) to exclude dead cells. Regulatory T cells were identified by endogenous Foxp3-tdTomato fluorescence (Foxp3IRES-tdTomato reporter mice, Shanghai Model Organisms Center, Inc., Stock NM-KI-190119), eliminating the need for intracellular Foxp3 antibody staining and thereby preserving native protein expression profiles. Treg cells were sorted as 7-AAD^−^CD4^+^Foxp3-tdTomato^+^ cells on a BD FACSAria III cell sorter (BD Biosciences).Total RNA was extracted from sorted Tregs using TRIzol reagent (Invitrogen, Cat. No. 15596026) and reverse-transcribed into cDNA using the PrimeScript RT Reagent Kit (Takara, Cat. No. RR037A). Quantitative real-time PCR (qRT-PCR) was performed on an ABI 7500 Real-Time PCR System (Applied Biosystems) using TB Green Premix Ex Taq II (Takara, Cat. No. RR820A). The following gene-specific primers were used: Ezh2 (forward: 5’-ACATCGAAGGCAGTGGAGTC-3’, reverse: 5’-CCAACAAACCGGTCCCTTCT-3’); Eed (forward: 5’-ATCCTCATAGCTGTCTGCGA-3’, reverse: 5’-CCTGATCGAATGCTGAAAAACA-3’); Cdkn2a (forward: 5’-GTACCCCGATTCAGGTGATG-3’, reverse: 5’-GGAGAAGGTAGTGGGGTCCT-3’); Cdkn1b (forward: 5’-AGCTGAGAGTGTCTAACGGGAG-3’, reverse: 5’-TTCATGATTGACCGGGCCGAAG-3’); and Suz12 (forward: 5’-CTCTGAGAACTTGCCCCATGT-3’, reverse: 5’-CAAGCAGACCCACAGAACCT-3’). Gapdh (forward: 5’-TGTCAAGCTCATTTCCTGGTATG-3’, reverse: 5’-GGGATGGAAATTGTGAGGGAGAT-3’) served as the internal control. Relative gene expression was calculated using the 2^-△△Ct^ method.

### Data acquisition

The data sources for this study encompassed scRNA-seq data and genome-wide association study (GWAS) data. The scRNA-seq datasets were downloaded from the European Genome-Phenome Archive (EGA) under accession number EGAS00001005271 from EBI/CRG (26). Specifically, the downloaded samples included skin tissue samples from eight patients with PV, seven patients with AD, and seven healthy controls. Subsequently, genetic data pertaining to PV and AD were retrieved from the Integrative Epidemiology Unit Open Genome-Wide Association Study (IEU OpenGWAS) database (https://gwas.mrcieu.ac.uk/). The GWAS dataset corresponding to PV was ebi-a-GCST90018907, comprising 483,174 samples (including 5,072 PV patients and 478,102 controls), encompassing 24,191,364 single nucleotide polymorphisms (SNPs). The GWAS dataset corresponding to AD was identified as ebi-a-GCST90018784, comprising 481,299 samples (including 6,224 AD patients and 475,075 controls), encompassing 24,185,642 SNPs. It is noteworthy that both GWAS datasets were sourced from European populations and were selected based on the principle of having the largest number of SNPs. Cis-eQTL summary statistics for the MR-screened genes were retrieved from the OpenGWAS database (eqtl-a-ENSG accessions) as instrumental variables. Detailed information on all GWAS outcome datasets and gene-specific cis-eQTL exposure datasets, including accession numbers, gene symbols, ENSEMBL IDs, sample sizes, and sources, is provided in **Supplementary Table 1**.

### Processing of scRNA-seq data

Quality control (QC) was performed on scRNA-seq datasets. The PercentageFeatureSet function from the Seurat package (v 5.0.1) (27) was utilized to filter cells and genes. All functions employed in this section were sourced from the Seurat package (v 5.0.1). The QC criteria were set as follows: cells with the number of detected genes (nFeature_RNA) greater than 200 and less than 6,000, and with a mitochondrial gene expression proportion (percent.mt) of less than 10% were retained. Following the filtration process, a dataset was constructed, comprising high-quality cells and genes. Subsequently, distinct Seurat sub-objects were generated for the quality-controlled data, which were then categorized into PV and AD groups. Following QC of the PV and AD scRNA-seq data, highly variable genes (HVGs) were further screened to minimize technical noise and highlight genuine biological differences among cells. The FindVariableFeatures function was employed to select the top 2,000 HVGs from the PV and AD scRNA-seq datasets, utilizing the variance stabilization transformation (VST) method. Simultaneously, the LabelPoints function was used to label the top 10 most variable genes. Afterwards, the NormalizeData and ScaleData functions were applied to normalize and standardize all samples in the PV and AD scRNA-seq datasets. Subsequently, with the 2,000 HVGs as input, principal component analysis (PCA) was conducted using the RunPCA function to reduce data dimensionality. The contribution of each gene to different principal components (PCs) was evaluated using the VizDimLoadings function. Subsequently, a scree plot was generated using the ElbowPlot function to determine an appropriate number of PCs. The JackStraw function was utilized to compute p-values for genes within each PC, enabling the identification of statistically significant PCs. Meanwhile, the ScoreJackStraw function quantified the significance strength of these components, with those containing a higher abundance of genes with low p-values being deemed more statistically meaningful. After obtaining high-quality PCs, unsupervised clustering analysis was conducted to further identify cell types. This was achieved by utilizing the FindNeighbors and FindClusters functions with a resolution parameter set to 0.4. Thereafter, the RunUMAP function was employed to visualize the clustering results based on the Uniform Manifold Approximation and Projection (UMAP) algorithm, thereby constructing the single-cell landscape for both the PV and AD groups. Subsequently, the FindAllMarkers function (with the following parameter settings: logfc.threshold = 0.25, min.pct = 0.1, only.pos = TRUE) was employed to identify specifically highly expressed genes in each cell cluster in the single-cell RNA sequencing datasets of PV and AD, respectively, based on the obtained clustered cell populations. The ggplot2 package (v 3.4.4) (28) was then used to generate heatmaps illustrating the average expression levels of the top three highly expressed genes in each cell cluster for the two pairs of groups (PV vs. control and AD vs. control), respectively. The cell type annotation for each cell cluster was conducted by integrating information from the literature (29, 30), with reference to the CellMarker database (http://biocc.hrbmu.edu.cn/CellMarker/) and the singleR package (v 2.4.0) (31). Subsequently, UMAP visualization was employed to display the annotated cell types for the comparisons of the PV group vs. the control group and the AD group vs. the control group. Furthermore, to compare differences in annotated cell types among different samples, the abundance of annotated cells was quantified separately in the scRNA-seq data of PV and AD. Stacked bar plots were then generated using the ggplot2 package (v 3.4.4) to illustrate the distribution of cell abundance between the PV and control groups, as well as between the AD and control groups. Following this, statistical analysis of the abundance differences for each annotated cell type between the two pairs of groups (PV vs. control and AD vs. control) was performed using the Wilcoxon test (P< 0.05).

### Dimensionality reduction clustering and annotation of T cells

Previous studies have revealed the proliferative expansion of Tregs and tissue-resident memory T cells (Trm), as well as widespread T-cell functional exhaustion, in inflammatory rashes (32). These studies also indicated that T-cell subpopulations are differentially dysregulated in AD and PV. Therefore, T cells were isolated separately and subjected to secondary dimensionality reduction and clustering to analyze, at a high level of detail, the composition and transcriptional characteristics of key subpopulations, such as Tregs. Specifically, based on the T-cell expression matrices of the PV group versus the control group and the AD group versus the control group, which were obtained from annotations, dimensionality reduction and clustering analyses were first conducted. Subsequently, UMAP clustering plots were generated to illustrate the distribution of T cells in the two pairs of groups (PV vs. control and AD vs. control). Subsequently, the FindAllMarkers function (with parameters set as logfc.threshold = 0.25, min.pct = 0.1, and only.pos = TRUE) from the Seurat package (v 5.0.1) was employed to screen for specifically highly expressed genes in each T-cell cluster, using PV-related scRNA-seq data and AD-related scRNA-seq data separately. The resulting gene lists were then annotated for subpopulations by integrating insights from relevant literature (33–35), along with the singleR package (v 2.4.0) and the CellMarker database. Thereafter, the RunUMAP function was utilized to generate distribution plots of the annotated T-cell subpopulations. Building on this, the abundance of major cell clusters across different samples was further quantified. Stacked bar plots were then created using the ggplot2 package (v 3.4.4), and the Wilcoxon test (P< 0.05) was applied to compare the abundance differences between the two pairs of groups (PV vs. control and AD vs. control).

### Pseudo-temporal trajectory analysis and enrichment analysis of T-cell subpopulations

The pseudo-temporal trajectory analysis was conducted on T-cell subpopulations associated with PV and AD utilizing monocle package (v 2.26.0) (36). Specifically, the FindNeighbors and FindClusters functions from the Seurat package (v 5.0.1) were first employed to conduct UMAP clustering and subsequent sub-clustering analysis of T cell subpopulations. Subsequently, the plot_cell_trajectory function from the monocle package (v 2.26.0) was utilized to construct a pseudo-temporal trajectory, by which cells were ordered according to their pseudo-time sequence and projected onto trajectory branches to simulate their developmental pathways. Additionally, Gene Ontology (GO) and Kyoto Encyclopedia of Genes and Genomes (KEGG) enrichment analyses (P< 0.05) were performed on Tregs from the control, PV, and AD groups using the clusterProfiler package (v 4.10.0) (37).

### Cellular communication analysis

To explore the interactions and signaling pathway characteristics between Treg subpopulations and other annotated immune cell types, cell communication analyses were conducted using the CellChat package (v 1.6.1) (38) based on scRNA-seq data from the PV group versus the control group and the AD group versus the control group, respectively. The analytical workflow involved constructing a network of ligands, receptors and associated factors to deduce communication relationships between different cell types, generating an intercellular interaction network diagram to illustrate connectivity between distinct cell types, and visualizing signal pathway input and output intensities via heatmaps to uncover the potential roles of various signaling pathways in intercellular communication.

### Differential gene expression analysis

Differential analyses were performed on scRNA-seq data from skin tissues of the PV group versus the control group and the AD group versus the control group to identify differentially expressed genes (DEGs) associated with Tregs. The FindMarkers function from the Seurat package (v 5.0.1) was used to identify DEGs. Genes with |average log_2_ fold change (FC)| > 0.25 and adjusted p-value< 0.05 were considered significant. Based on these criteria, DEGs between the PV and control groups, as well as between the AD and control groups, were identified and designated as PV-DEGs and AD-DEGs, respectively. Subsequently, the ggplot2 package (v 3.4.4) was utilized to create volcano plots for PV-DEGs and AD-DEGs, with the top 10 up-regulated and top 10 down-regulated genes labeled (ranked based on their average log_2_FC values).

### Functional enrichment analysis and protein-protein interaction network construction of candidate genes

To further screen for DEGs related to Tregs that were commonly present in both PV and AD, the VennDiagram package (v 1.7.3) (39) was utilized to conduct an intersection analysis on the previously obtained PV-DEGs and AD-DEGs. The shared genes were then defined as candidate genes. Subsequently, the clusterProfiler package (v 4.10.0) (40) was employed to carry out GO enrichment analysis on the candidate genes (adjusted p-value< 0.05). This analysis shed light on the relevant molecular functions (MFs), cellular components (CCs), and biological processes (BPs) linked to these genes. Concurrently, the same package was used to perform KEGG enrichment analysis (p-value< 0.05), to pinpoint crucial signaling pathways associated with the candidate genes. To explore the interactions between candidate genes at the protein level, the genes were entered into the Search Tool for the Retrieval of Interacting Genes (STRING) database (https://string-db.org/) using a confidence score threshold of ≥ 0.4 and a maximum input of 2,000 genes. Thereafter, Cytoscape software (v 3.10.2) (41) was used to visualize the PPI network for the corresponding proteins of genes with a degree of connectivity of at least 100.

### Selection of instrumental variables

To further explore the causal associations between candidate genes and PV, as well as between candidate genes and AD, the candidate genes were designated as the exposure factor, and PV and AD were considered the outcomes. Two-sample Mendelian randomization (MR) analyses were then conducted separately for the relationships between the candidate genes and PV, and between the candidate genes and AD. The MR analysis was based on three fundamental principles. Firstly, the IVs considered had to demonstrate substantial statistical associations with the exposure of interest. Secondly, the chosen genetic variants had to be free from any potential confounders. Thirdly, these variants had to affect the outcome solely via the exposure, without involving any alternative mediating pathways. In this research, the extract_instruments function from the TwoSampleMR package (v 0.6.4) (42) was utilized to screen for IVs significantly associated with the candidate genes. The screening criteria were as follows: (1) A significance threshold of p-value< 5 × 10^-8^ was applied to identify SNPs significantly associated with the candidate genes. (2) Loci in linkage disequilibrium were eliminated using the parameter clump = TRUE (R² = 0.001, distance = 10,000 kb). (3) SNPs significantly associated with the outcome, as determined by outcome GWAS data, were excluded (proxies = TRUE, r² = 0.8). (4) The IVs (SNPs) underwent F-statistic tests were performed as follows: $\text{F}=(\frac{\text{n}-\text{k}-1}{\text{k}})(\frac{{\text{R}}^{2}}{1-{\text{R}}^{2}})$. The “R²” value represented the squared correlation coefficient between the IVs (SNPs) and the candidate genes, where “n” indicated the sample size and “k” denoted the number of IVs (SNPs). IVs with an F-statistic greater than 10 were considered valid. (5) It was ensured that the number of valid SNPs exceeded two. The strength of all genetic instruments was assessed by calculating F-statistics for each SNP-exposure association. F-statistics ranged from 29.72 to 6426.52 across all instrumental variables, substantially exceeding the conventional threshold of 10 and indicating negligible weak instrument bias. Detailed F-statistics for each gene are provided in **Supplementary Table 2**.

### Two-sample MR analysis

Causal associations between candidate genes and PV, as well as between candidate genes and AD, were investigated utilizing the TwoSampleMR package (v 0.6.4) alongside five algorithms: MR Egger (43), weighted median (44), inverse variance weighted (IVW) (45), simple mode (46), and weighted mode (47). The IVW method was employed as the main analytical technique. A statistically significant result (p-value< 0.05) suggested evidence of a causal link between candidate genes and PV/AD. Furthermore, scatter and forest plots were created using the mr_scatter_plot and mr_forest_plot functions, respectively, from the TwoSampleMR package (v 0.6.4) for visualizing the MR findings. The mr_funnel_plot function from the same package was also used to generate funnel plots to assess compliance with Mendel’s second law.

### Sensitivity analysis

Sensitivity analysis was conducted using the TwoSampleMR package (v 0.6.4) to verify the robustness and reliability of the MR analysis results. Firstly, the heterogeneity test was conducted on the IVs using Cochran’s Q statistic, with the aim of evaluating the consistency of effect estimates across multiple SNPs. If the test yielded a p-value > 0.05, this indicated that there was no significant heterogeneity among the IVs, and a fixed-effects model (e.g. mr_ivw_fe) was applied for the subsequent analysis. Conversely, if the p-value ≤ 0.05, it suggested significant heterogeneity and a random-effects model (e.g. mr_ivw_mre) was more appropriate to account for differences in the effects of the IVs. Secondly, MR-Egger regression and MR-PRESSO methods were employed to analyze whether the IVs influenced the outcomes through pathways other than the exposure factor (i.e. to assess the presence of horizontal pleiotropy). If the P-values from the MR-Egger or MR-PRESSO tests were greater than 0.05, it was concluded that there was no significant horizontal pleiotropy and that the results of the causal inference were more reliable. Thirdly, the mr_leaveoneout function from the TwoSampleMR package (v 0.6.4) was utilized to conduct leave-one-out (LOO) analysis. This involved sequentially excluding each IV (SNP) to observe its impact on the causal effect estimation and assess whether any single SNP significantly biased the overall results. If the overall effect estimate remained stable throughout the sequential exclusion process, the MR results were considered to exhibit good reliability. Finally, to verify that the MR results were not confounded by reverse causality, the steiger_filtering function from the TwoSampleMR package (v 0.6.4) was employed to conduct Steiger’s test for reverse causation. When Steiger-direction was TRUE and the p-value was statistically significant (P< 0.05), it indicated that the causal direction between the exposure and outcome was correctly identified. Steiger’s directionality test confirmed that the causal direction (gene expression → disease risk) was correctly specified for all significant gene-disease pairs (Steiger direction = TRUE, P< 0.05 for all tests). No ambiguous directionality was detected. Detailed Steiger test statistics are reported in **Supplementary Table 3**. Candidate genes identified via MR analysis and sensitivity analysis, with PV serving as the outcome, were classified as PV-feature genes. Likewise, candidate genes determined through the same analytical approaches but with AD as the outcome were designated as AD-feature genes.

### Identification and functional analysis of co-expressed genes

An intersection analysis was conducted on PV-feature genes and AD-feature genes using the VennDiagram package (v 1.7.3) to identify Treg-related signature genes that were causally linked and co-expressed in both PV and AD. These genes were designated as co-expressed genes. Thereafter, GO and KEGG enrichment analyses (p-value< 0.05) were performed on these co-expressed genes using the clusterProfiler package (v 4.10.0) to investigate their involvement in BPs, CCs, MFs, as well as relevant metabolic and signaling pathways. To further investigate the protein-level interactions of the co-expressed genes, the genes were imported into the STRING database (confidence score ≥ 0.15), and the resulting PPI network was visualized using Cytoscape software (3.10.2).

### Gene set enrichment analysis

To further elucidate the potential functions and signaling pathways of co-expressed genes in different diseases, GSEA was conducted. Initially, based on the Molecular Signatures Database (MSigDB) (https://www.gsea-msigdb.org/), “c2.cp.kegg.v7.4.symbols.gmt” was selected as the reference gene set. Using the psych package (v 2.4.3) (https://CRAN.R-project.org/package=psych), the correlation between each co-expressed gene and the remaining genes was calculated, and the genes were then ranked in descending order according to the correlation coefficients, thereby generating a gene list for each co-expressed gene. Subsequently, GSEA was performed on each co-expressed gene using the package clusterProfiler (v 4.10.0), with the screening criteria set as |normalized enrichment score (NES)| > 1, false discovery rate (FDR)< 0.05, and p-value< 0.05.

### Construction of regulatory networks

Further uncovering the potential mechanisms of action of co-expressed genes at the levels of transcriptional regulation and non-coding RNA regulation, this study constructed transcription factor (TF)-co-expressed gene regulatory network and long non-coding RNA (lncRNA)–microRNA (miRNA)–co-expressed gene regulatory networks, respectively. At the TF level, the ChEA3 database (https://maayanlab.cloud/chea3/) was utilized to predict TFs that exhibited regulatory relationships with co-expressed genes, and the results were visualized using Cytoscape software (v 3.10.2) to elucidate the potential transcriptional regulatory patterns governing co-expressed genes. At the level of non-coding RNA, potential miRNAs targeting co-expressed genes were predicted based on the TargetScan database (https://www.targetscan.org/vert_80/) and miRWalk database (http://mirwalk.umm.uni-heidelberg.de/) separately, and the intersection was taken to ensure result reliability. Subsequently, related lncRNAs were predicted from the StarBase database (https://rnasysu.com/encori/) using a screening threshold of clipExpNum > 20. Finally, the lncRNA–miRNA–mRNA regulatory relationships were integrated and visualized as a Sankey diagram using the ggsankey package (v 0.0.9) (48).

### Drug/compound prediction and molecular docking

To further explore potential therapeutic drugs/compounds targeting co-expressed genes, this study was conducted based on drug/compound prediction and molecular docking analysis. Specifically, the enrichR package (v 3.2) (49) was employed to conduct drug/compound-association searches for the co-expressed genes using the Comparative Toxicogenomics Database (CTD) (http://ctdbase.org/). Compounds with a reference count ≥ 2 were filtered to obtain candidate drugs/compounds significantly associated with the co-expressed genes, and the ggsankey package (v 0.0.9) was utilized to generate a drug/compound–gene relationship diagram. For each co-expressed gene, the drug achieving the highest overall score was chosen as the candidate drug/compound. In molecular docking, the 3D structures of candidate drugs/compounds were retrieved from the public chemistry database (PubChem) database (https://pubchem.ncbi.nlm.nih.gov/), and the structural information of corresponding co-expressed gene proteins was downloaded from the AlphaFold Protein Structure Database (AlphaFold) (https://alphafold.ebi.ac.uk/). Subsequently, molecular docking simulations were performed using the CB-Dock platform (https://cadd.labshare.cn/cb-dock2/php/index.php), with binding free energy serving as the evaluation metric for the interaction strength between drugs/compounds and target co-expressed genes.

### Expression differences of co-expressed genes in Treg subsets

The Wilcoxon test was employed to conduct differential expression analysis of co-expressed genes in various Treg subsets from both PV and AD patients (p-value< 0.05). The objective of this approach was to statistically evaluate the expression differences of these genes among distinct Treg subsets (cmTreg, eTreg, and Treg-c), thereby uncovering their differential roles in varying immune microenvironments.

### Statistical analysis

Statistical analyses were conducted using R (v4.3.1), GraphPad Prism 9.0, and FlowJo 10.8 for flow cytometry data analysis. All data are expressed as mean ± standard error of the mean (SEM). Differences between two groups were assessed using the unpaired Student’s t-test. For bioinformatics analyses, statistical significance was set at P< 0.05 unless otherwise specified.

## Results

### Single-cell profiling and animal model validation reveal shared Treg subset remodeling in PV and AD

**Figure 1:**
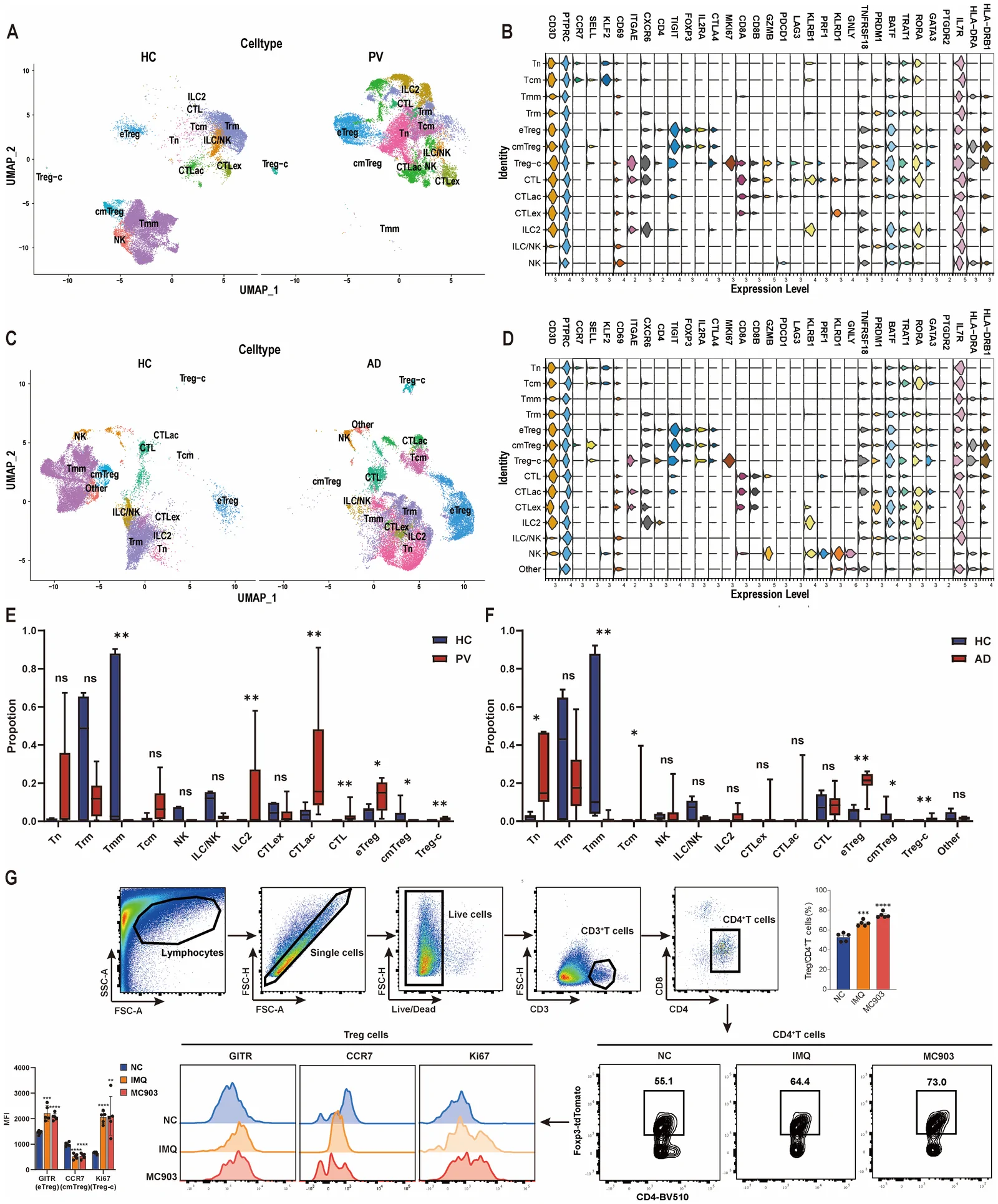
scRNA-seq reveals altered T cell subpopulation landscapes in PV and AD and validation in murine models. **(A)** UMAP plot of 13 T cell subclusters from HC and PV samples, including Tn, Tcm, Tmm, Trm, cmTreg, eTreg, Treg-c, CTL, CTLac, CTLex, ILC2, ILC/NK, and NK cells. **(B)** Violin plots showing canonical marker gene expression across 13 T cell subclusters from HC and PV samples, including CD3D, PTPRC, SELL, KLF2, CD69, ITGAE, CXCR6, CD4, TIGIT, FOXP3, IL2RA, CTLA4, MKI67, CD8A, CD8B, GZMB, PDCD1, LAG3, KLRB1, PRF1, KLRD1, GNLY, TNFRSF18, PRDM1, BATF, TRAT1, RORA, GATA3, PTGDR2, IL7R, HLA-DRA, and HLA-DRB1. **(C)** UMAP plot of 13 T cell subclusters plus an “Other” cluster from HC and AD samples. **(D)** Violin plots showing marker gene expression across 13 T cell subclusters and the “Other” cluster from HC and AD samples. **(E)** Box plots comparing T cell subpopulation proportions between PV and HC samples, with statistical significance indicated. **(F)** Box plots comparing T cell subpopulation proportions between AD and HC samples, with statistical significance indicated. **(G)** Flow cytometry gating strategy for Foxp3-tdTomato reporter mice, representative Foxp3-tdTomato flow cytometry plots in CD4^+^T cells and quantification, MFI of GITR, CCR7, and Ki67 in Treg cells with representative histograms and quantification across NC, IMQ, and MC903 groups (n = 5). Data are presented as mean ± SEM. Statistical significance was determined by unpaired Student’s t-test (*P < 0.05, **P < 0.01, ***P < 0.001, ****P < 0.0001, *ns*, not significant).

We obtained publicly available scRNA-seq data of lesional skin from 7 healthy donors (HC), 8 patients with psoriasis vulgaris (PV), and 7 patients with atopic dermatitis (AD) (accession number EGAS00001005271 at EBI/CRG). After quality control, single-cell transcriptomic profiling revealed significant gene expression differences in PV and AD compared with controls (**Supplementary Figure 1A**). T lymphocytes constituted the most abundant cell type in both diseases, with significantly elevated relative proportions compared with controls (**Supplementary Figures 1B–D**), underscoring the central involvement of T cell-mediated immunopathology. To delineate the disease-specific subpopulation architecture of T cells, we performed unsupervised reclustering of T cells from scRNA-seq data. This analysis resolved 28 transcriptionally distinct subclusters in PV and 30 subclusters in AD (**Supplementary Figures 2A, C**), which were further annotated into 13 shared subpopulations: naive T cell (Tn), central memory T cell (Tcm), migratory memory T cell (Tmm), tissue-resident memory T cell (Trm), central memory Treg (cmTreg), effector Treg (eTreg), cycling Treg (Treg-c), cytotoxic T lymphocyte (CTL), activated CD8^+^ T cell (CTLac), exhausted CD8^+^ T cell (CTLex), innate lymphoid cell 2 (ILC2), ILC/NK, and NK (**Figures 1A–D**).

Among these, quantitative comparison between the PV and AD groups versus controls revealed dramatic alterations in Treg subset distribution (**Figures 1E, F**; **Supplementary Figures 2B, D**; **Tables 1**, **2**); therefore, three transcriptionally distinct Treg subsets emerged as the focal point of subsequent analysis. The cmTreg subset displayed a lymphoid-homing signature defined by SELL (CD62L), CCR7, and the memory-associated CD45RO isoform, consistent with a central memory-like phenotype that may contribute to systemic immune surveillance through secondary lymphoid organs (26, 50). The eTreg subset expressed elevated levels of effector molecules TNFRSF18 (GITR) and PRDM1, suggesting a role in tissue-adapted effector-phase immunosuppression (26). The Treg-c subset exhibited a pronounced proliferative transcriptional program marked by MKI67, TOP2A, CENPF, and UBE2C (26), indicative of active cell cycle engagement and clonal expansion (**Figures 1B, D**). Quantitative analysis revealed a shared remodeling pattern in both diseases. In PV lesions, cmTreg was nearly absent (828 cells in HC vs. 5 in PV), while Treg-c (7 vs. 215) and eTreg (547 vs. 3578) were markedly expanded (**Figure 1E**; **Table 1**). AD lesions showed the same directional changes: cmTreg was reduced (784 vs. 2), Treg-c expanded (7 vs. 375), and eTreg increased (542 vs. 4911) (**Figure 1F**; **Table 2**). This cross-disease concordance indicates that cmTreg depletion coupled with Treg-c and eTreg expansion is a shared pathogenic feature of PV and AD. The near-total loss of cmTreg suggests a disruption of the long-lived regulatory reservoir that may normally sustain skin immune tolerance through lymphoid tissue-based renewal, whereas the expansion of Treg-c likely reflects a compensatory proliferative response by the residual regulatory compartment. The disproportion between cmTreg loss (823 cells in PV, 782 in AD) and Treg-c gain (208 in PV, 368 in AD) further suggests that the compensatory proliferation is quantitatively insufficient to restore regulatory homeostasis (**Tables 1**, **2**).

**Table 1:** Quantitative distribution of distinct T cell subsets between the PV group and the control group.

Group	NK	ILC/NK	ILC2	CTLex	CTLac	CTL	Treg-c	cmTreg	eTreg	Trm	Tmm	Tcm	Tn
CK	792	1111	9	572	433	3	7	828	547	4974	10534	139	86
PV	8	398	2548	1046	4477	791	215	5	3578	2448	13	2174	4541

**Table 2:** Quantitative distribution of distinct T cell subsets between the AD group and the control group.

Group	Other	NK	ILC/NK	ILC2	CTLex	CTLac	CTL	Treg-c	cmTreg	eTreg	Trm	Tmm	Tcm	Tn
CK	563	477	774	25	2	3	947	7	784	542	4894	10917	4	236
AD	318	376	304	489	110	1253	1643	375	2	4911	5027	318	1447	5934

To validate these human lesional findings, we employed Foxp3-tdTomato reporter mice and established two disease models: imiquimod (IMQ)-induced psoriasis-like dermatitis (51) and MC903-induced atopic dermatitis (52). Consistent with the human lesional scRNA-seq data, diseased mouse skin exhibited increased total Treg infiltration. Subpopulation analysis confirmed the identical remodeling signature: characteristic markers of eTreg (GITR) and Treg-c (Ki67) were significantly expanded, whereas the cmTreg marker (CCR7) was markedly reduced (**Figure 1G**). These results corroborate the human findings and indicate that Treg subset remodeling pattern is a conserved feature of cutaneous inflammation in both species.

### Pseudo-temporal trajectory and cellular communication analysis revealed dynamic differentiation and remodeling of Treg subsets in PV and AD

**Figure 2:**
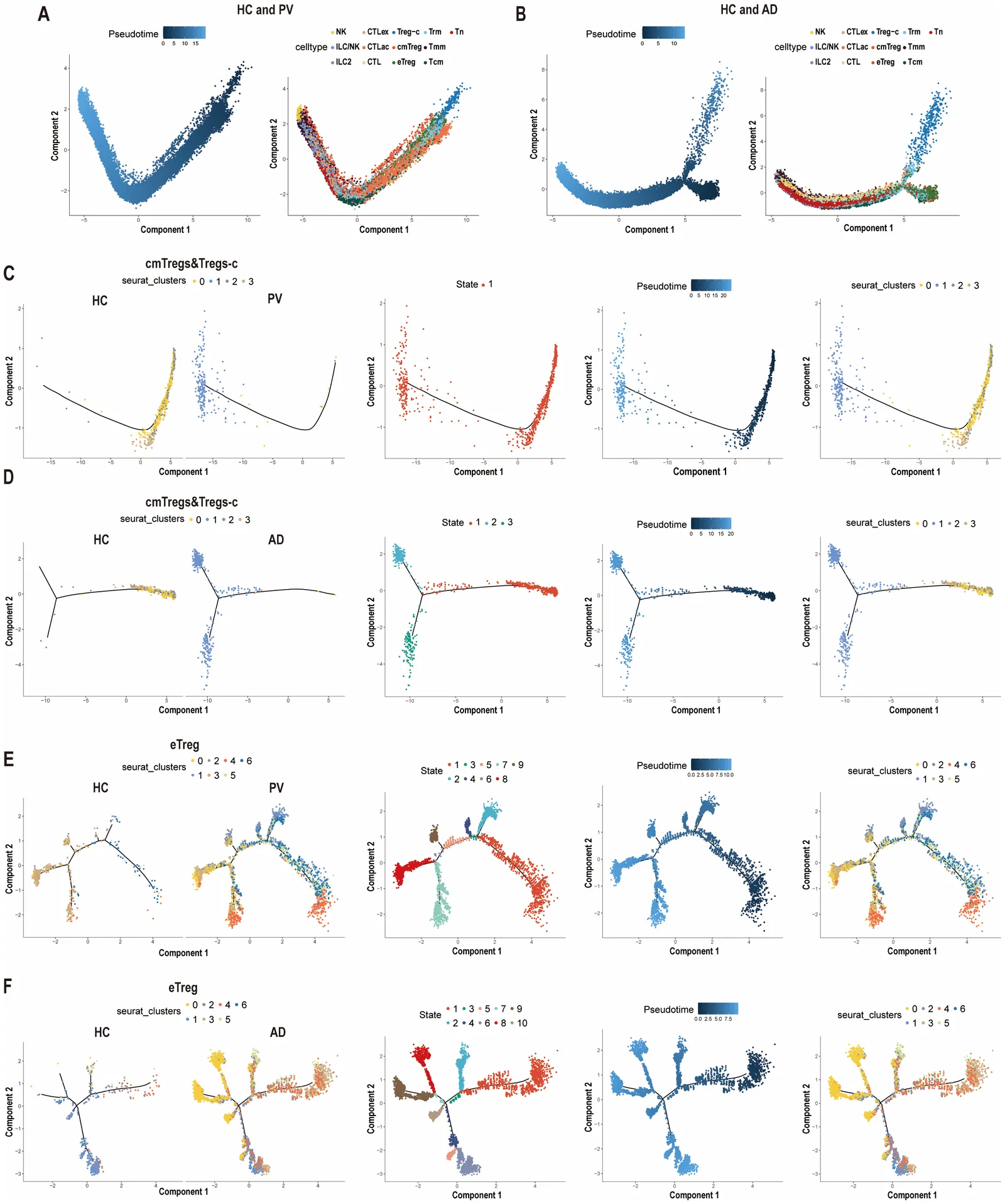
Pseudotemporal trajectory analysis reveals dynamic differentiation patterns of T cell subpopulations in PV and AD. **(A)** Pseudotime trajectory plot of T cells from PV and HC samples, colored by cell subtype. **(B)** Pseudotime trajectory plot of T cells from AD and HC samples, colored by cell subtype. **(C)** Pseudotime trajectory of combined cmTreg and Treg-c populations in HC and PV samples. **(D)** Pseudotime trajectory of combined cmTreg and Treg-c populations in HC and AD samples. **(E)** Pseudotime trajectory of eTreg populations in HC and PV samples. **(F)** Pseudotime trajectory of eTreg populations in HC and AD samples.

We first performed pseudotime trajectory analysis on all T cell subpopulations to obtain an overview of differentiation dynamics. This analysis revealed that memory T cell subpopulations (Tcm, Tmm, Trm) and Treg subsets (cmTreg, Treg-c, eTreg) all demonstrated progressive differentiation along the pseudotime axis (**Figures 2A, B**), reflecting their roles and dynamic changes at different stages of the immune response. To further investigate the dynamic changes in Tregs under disease conditions, we conducted a pseudotime analysis restricted to the three Treg subsets. Owing to the limited abundance of cmTreg and Treg-c in each group, these two subsets were combined into cmTreg&Treg-c for subsequent analysis. The combined cmTreg&Treg-c population could be divided into four subclusters (**Figures 2C, D**). In PV and AD, cmTreg&Treg-c was predominantly distributed at the later stages of pseudotime, whereas in controls these cells were concentrated at early stages, suggesting that the cmTreg&Treg-c compartment in both diseases had shifted toward a more differentiated state (**Figures 2C, D**). Meanwhile, eTreg was subdivided into seven subclusters (**Figures 2E, F**). In controls, eTreg was concentrated at the terminal stage of pseudotime, indicating completed mature differentiation with stable suppressive function. In contrast, eTreg in PV and AD exhibited a broader and more even distribution along the pseudotime axis, without forming a concentrated terminal distribution. This dispersal suggests that eTreg in both diseases appeared not to concentrate at terminal maturation stages, potentially indicating incomplete effector differentiation that may compromise their capacity to maintain immune homeostasis and contain inflammatory cascades. Taken together, these pseudotime findings reveal a fundamental defect in Treg differentiation coordination in both PV and AD: the cmTreg&Treg-c compartment is prematurely driven toward advanced differentiation stages, yet the eTreg population fails to complete terminal maturation and instead disperses across immature stages. This disruption of the normal differentiation sequence suggests that Treg suppressive deficiency in PV and AD stems not merely from quantitative subset imbalance, but from a qualitative failure to produce fully competent effector regulatory cells.

**Figure 3:**
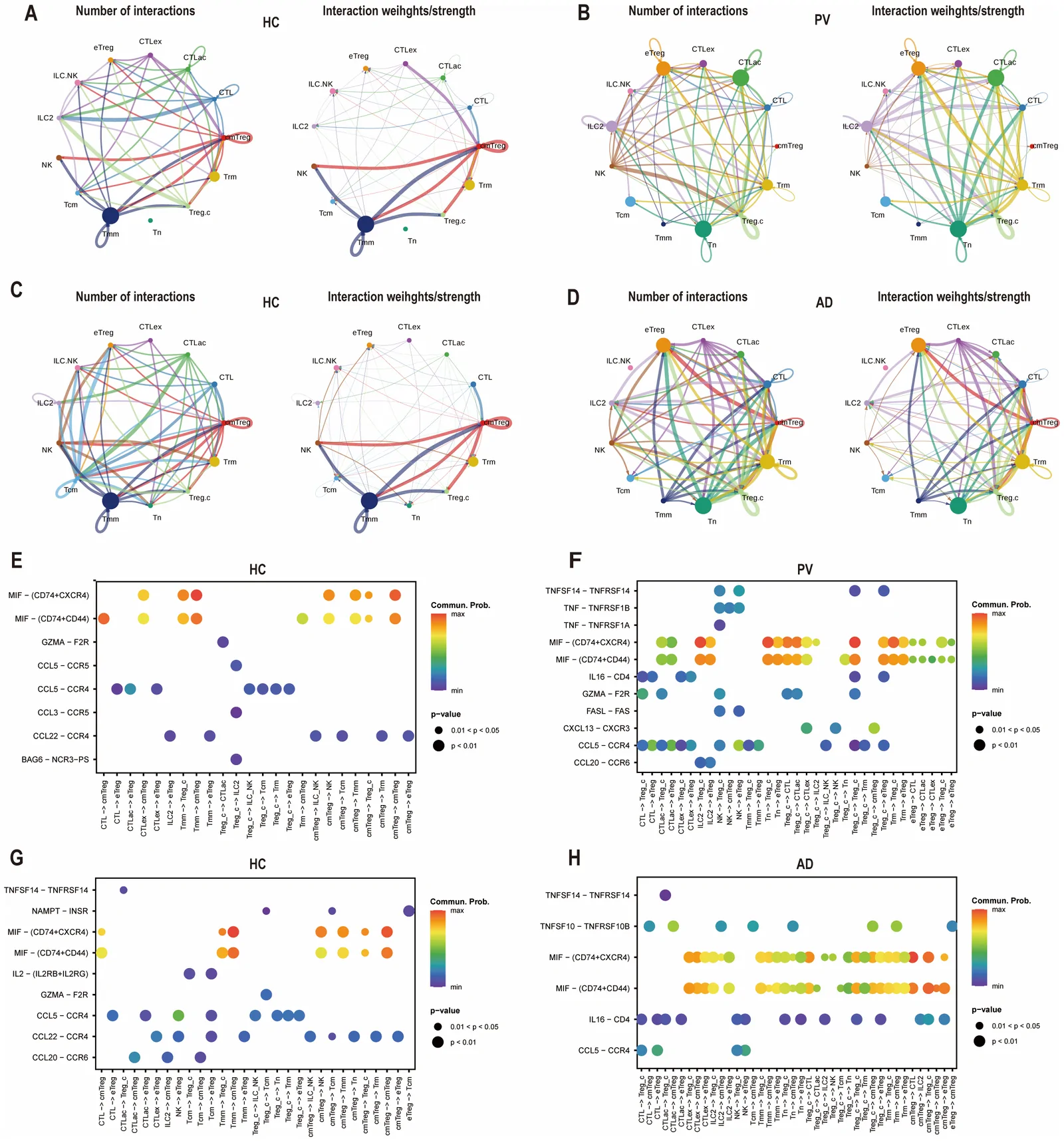
Cell-cell communication analysis reveals distinct intercellular interactions and dysregulated ligand-receptor interactions in PV and AD. **(A)** Cell-cell communication network plots for the HC group from the PV dataset, showing the number of interactions (left) and interaction weights/strength (right) among T cell subpopulations. **(B)** Cell-cell communication network plots for the PV group. **(C)** Cell-cell communication network plots for the HC group from the AD dataset. **(D)** Cell-cell communication network plots for the AD group. **(E)** Bubble plot showing enriched ligand-receptor pairs in the HC group from the PV dataset, with communication probability (Comm.Prob.) indicated by color and P value by bubble size. **(F)** Bubble plot showing enriched ligand-receptor pairs in the PV group. **(G)** Bubble plot showing enriched ligand-receptor pairs in the HC group from the AD dataset. **(H)** Bubble plot showing enriched ligand-receptor pairs in the AD group.

In terms of cellular communication, PV and AD exhibited distinct remodeling of intercellular signaling networks when compared to the control group. In the control samples, intercellular communications were relatively concentrated, with cmTregs acting as the major interaction hub and maintaining strong signaling with multiple immune cell subsets, including Trm, Treg-c and NK (**Figures 3A, C**), suggesting a stable and coordinated immune regulatory network. In contrast, both PV and AD exhibited increased numbers and intensities of intercellular communications, accompanied by a redistribution of interaction centers among Treg subsets (**Figures 3B, D**). cmTreg-mediated interactions were reduced, whereas Tregs-c and eTregs displayed enhanced crosstalk with various immune cells, particularly CTLs, Trms, and ILC2s. These findings indicate that in both diseases, the Treg communication network shifts from a cmTreg-centric topology to one jointly centered on Treg-c and eTreg.

Ligand-receptor pair analysis further revealed that specific signaling pathways between multiple immune cell subsets were upregulated in the disease groups compared with the control group (**Figures 3E–H**). Interactions involving MIF-(CD74+CXCR4/CD44) were significantly increased in both PV and AD compared with controls, although the signal transmission patterns differed between the two diseases. MIF signaling has been implicated in various inflammatory conditions through its pleiotropic pro-inflammatory effects (53–55). In the PV group, cmTregs almost entirely exited their role as MIF signal senders, whereas Tregs-c became the core signal senders, transmitting MIF signals to CTL, CTLac, CTLex and multiple Treg subsets; eTregs primarily served as signal receivers (**Supplementary Figure 3A**). In the AD group, a similar pattern was observed, with Tregs-c sending MIF signals to various cell types while eTregs mainly functioned as signal receivers (**Supplementary Figure 3B**). Additionally, novel signaling pathways emerged in PV, including TNF-(TNFRSF1B/TNFRSF1A), FASL-FAS, and CXCL13-CXCR3, whereas AD uniquely exhibited TNFSF10-TNFRSF10B and IL16-CD4 signaling pathways (**Supplementary Figure 3C**). Collectively, these results demonstrated that PV and AD share a common pattern of MIF signaling pathway remodeling, characterized by attenuated cmTreg-centered MIF signal transmission, enhanced Tregs-c-mediated MIF signal delivery, and eTregs serving as the predominant signal receivers.

### Distinct pathway enrichment profiles reveal disease-associated remodeling of Treg subsets in PV and AD

**Figure 4:**
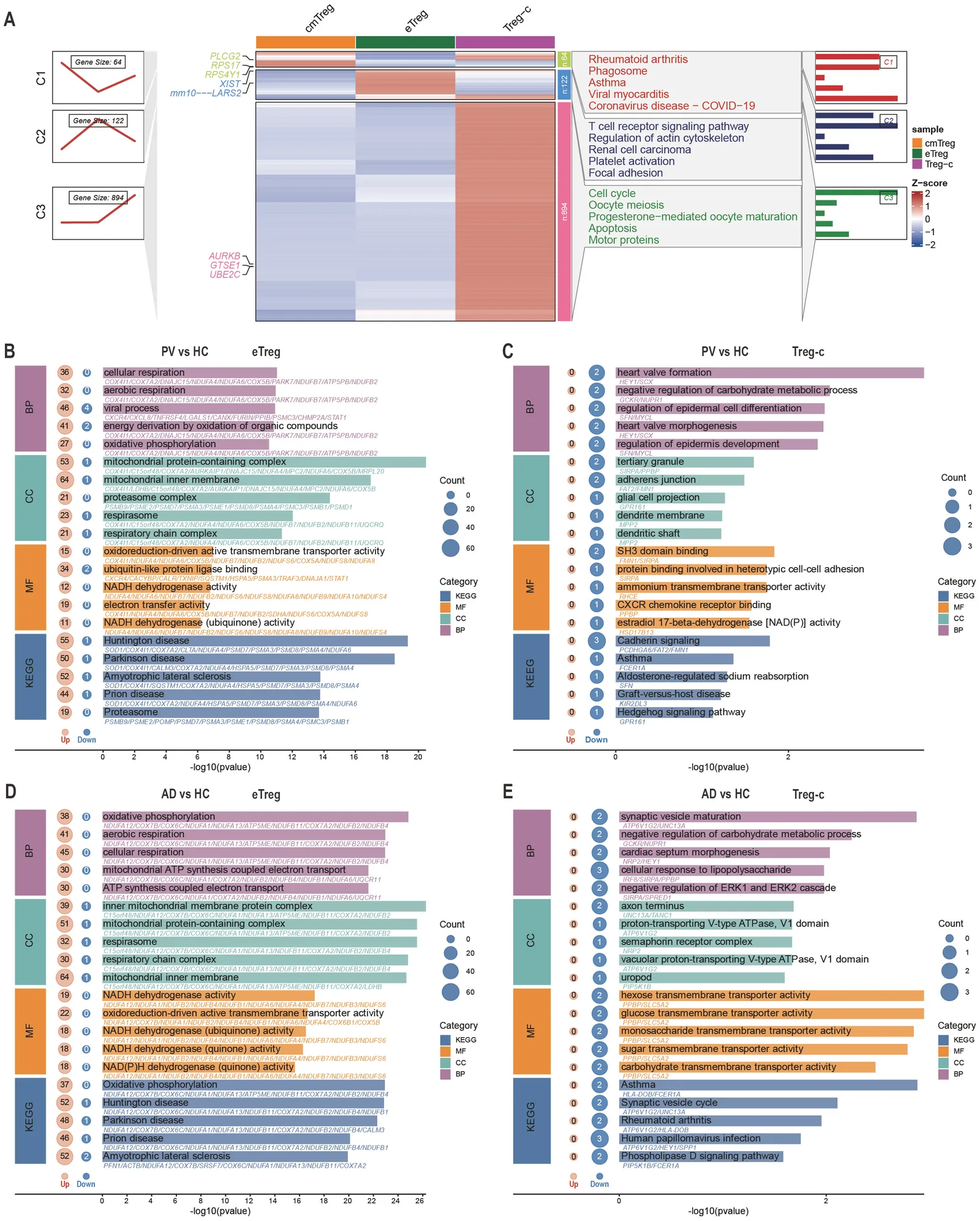
Functional enrichment analysis reveals distinct pathway signatures of Treg subsets in PV and AD. **(A)** KEGG enrichment heatmap and corresponding pathway bar plots for cmTreg, eTreg, and Treg-c subclusters from HC samples. **(B)** GO and KEGG enrichment analyses of differentially expressed genes (DEGs) in eTreg cells between PV and HC. **(C)** GO and KEGG enrichment analyses of DEGs in Treg-c cells between PV and HC. **(D)** GO and KEGG enrichment analyses of DEGs in eTreg cells between AD and HC. **(E)** GO and KEGG enrichment analyses of DEGs in Treg-c cells between AD and HC.

To elucidate the baseline characteristics of Treg subsets in healthy skin, KEGG enrichment analysis was performed on all marker genes of cmTregs, eTregs, and Tregs-c from normal control samples (**Figure 4A**). Heatmap clustering analysis identified three major gene clusters: Cluster C1 contained PLCG2 and ribosomal protein genes (RPS17, RPS4Y1), which were primarily enriched in the Phagosome pathway. These marker genes showed higher enrichment in cmTregs, suggesting a pivotal role for phagosome-mediated processes in the maintenance of the central memory regulatory pool. Cluster C2 was represented by XIST and interferon-stimulated genes LARS2, enriched in pathways including T cell receptor signaling, regulation of actin cytoskeleton, platelet activation, and focal adhesion. These genes and pathways were predominantly enriched in eTregs, reflecting the active functions of this subset in TCR-mediated activation, cytoskeletal dynamics, and tissue migration adhesion. Cluster C3, marked by cell cycle genes (AURKB, GTSE1, UBE2C), showed the most pronounced subset-specific enrichment, with Cell cycle and Motor protein pathways concentrated in Treg-c. This enrichment pattern indicates that Treg-c is the primary proliferative compartment of the Treg lineage under homeostatic conditions, and its active proliferation continuously supplies new cells to replenish the Treg population.

Having established the baseline characteristics of Treg subsets, we next analyzed their disease-associated pathway enrichment profiles under disease conditions. Because cmTregs were almost completely depleted in diseased skin, we focused our analysis on eTreg and Tregs-c functional alterations. Compared with normal controls, PV and AD exhibited a highly concordant eTreg differential gene enrichment profile; both diseases showed significant enrichment in mitochondrial respiratory chain and oxidative phosphorylation pathways, with enriched genes predominantly upregulated (**Figures 4B, D**; **Supplementary Table 4**). Treg metabolic reprogramming has been increasingly recognized as a critical determinant of suppressive function in inflammatory microenvironments (56, 57). This augmented metabolic activity may reflect an attempt by eTregs to maintain or compensate for their immunosuppressive function through increased energy supply in the inflammatory microenvironment; however, sustained metabolic stress may also lead to eTreg functional exhaustion or insufficient suppressive activity, thereby failing to effectively control local inflammatory responses. Subsequent analysis of Treg-c pathway enrichment under disease conditions revealed that all enriched genes in the Treg-c differential enrichment profiles of both PV and AD were downregulated, with disease-specific differences in the downregulated pathways (**Figures 4C, E**; **Supplementary Table 5**). In PV, significant downregulation of Tregs-c in epidermal differentiation and cadherin adhesion signaling pathways suggests impaired capacity for maintaining epidermal barrier homeostasis under disease conditions. In AD, significant downregulation of Tregs-c in carbohydrate transmembrane transport activity and LPS response pathways suggests diminished capability in meeting energy metabolic demands and regulating innate immune inflammatory responses.

In summary, eTreg mounts a compensatory mitochondrial metabolism response in an attempt to sustain suppressive capacity in both diseases, whereas Treg-c shows disease-specific functional decline. Together with cmTreg depletion, these subset-level alterations collectively disrupt immune tolerance in PV and AD.

### scRNA-seq screening and MR identify shared Treg-related candidate genes in PV and AD

The disease-associated pathway alterations observed in Treg subsets are driven by differential gene expression. To identify the specific genes underlying these shared pathological changes, we performed differential expression analysis on Treg cells from PV and AD scRNA-seq data. This analysis identified 3,162 PV-DEGs (3,038 up-regulated, 124 down-regulated) (**Supplementary Figure 4A**) and 2,829 AD-DEGs (2,699 up-regulated, 130 down-regulated) (**Supplementary Figure 4B**). The intersection of these two gene sets yielded 2385 candidate genes shared between PV and AD (**Supplementary Figure 4C**). GO enrichment analysis revealed that these candidate genes were significantly enriched in biological processes such as RNA splicing, nucleocytoplasmic transport, and proteasome-mediated protein catabolism; at the cellular component level, they localized to chromosomal regions, nuclear speckles, and SWI/SNF chromatin remodeling complexes; molecular function analysis highlighted histone binding, histone modifying activity, and transcription coactivator activity (**Supplementary Table 6**; **Supplementary Figure 4D**). KEGG pathway analysis further identified enrichment in T cell receptor signaling, ATP-dependent chromatin remodeling, proteasome and ubiquitin-mediated proteolysis, and ribosome biogenesis, alongside multiple viral infection response pathways (**Supplementary Table 6**; **Supplementary Figure 4E**). These convergent enrichment patterns indicate that the shared candidate genes between PV and AD converge on three core biological axes: epigenetic regulation (chromatin remodeling and histone modification), T cell activation and signaling (TCR signaling and nucleocytoplasmic transport), and protein homeostasis (proteasome-mediated degradation and ribosomal function), collectively implicating dysregulated transcriptional control and immune signaling as central mechanisms underlying Treg dysfunction in both diseases. In the PPI network analysis, the top 2,000 candidate genes were entered, and after removing isolated gene nodes, proteins corresponding to 93 candidate genes were obtained, among which AKT1 emerged as a core node with a high degree of connectivity (**Supplementary Figure 4F**), indicating that AKT1 may play a central regulatory role within the interaction network and participate in key signaling pathways involved in the progression of both diseases.

**Figure 5:**
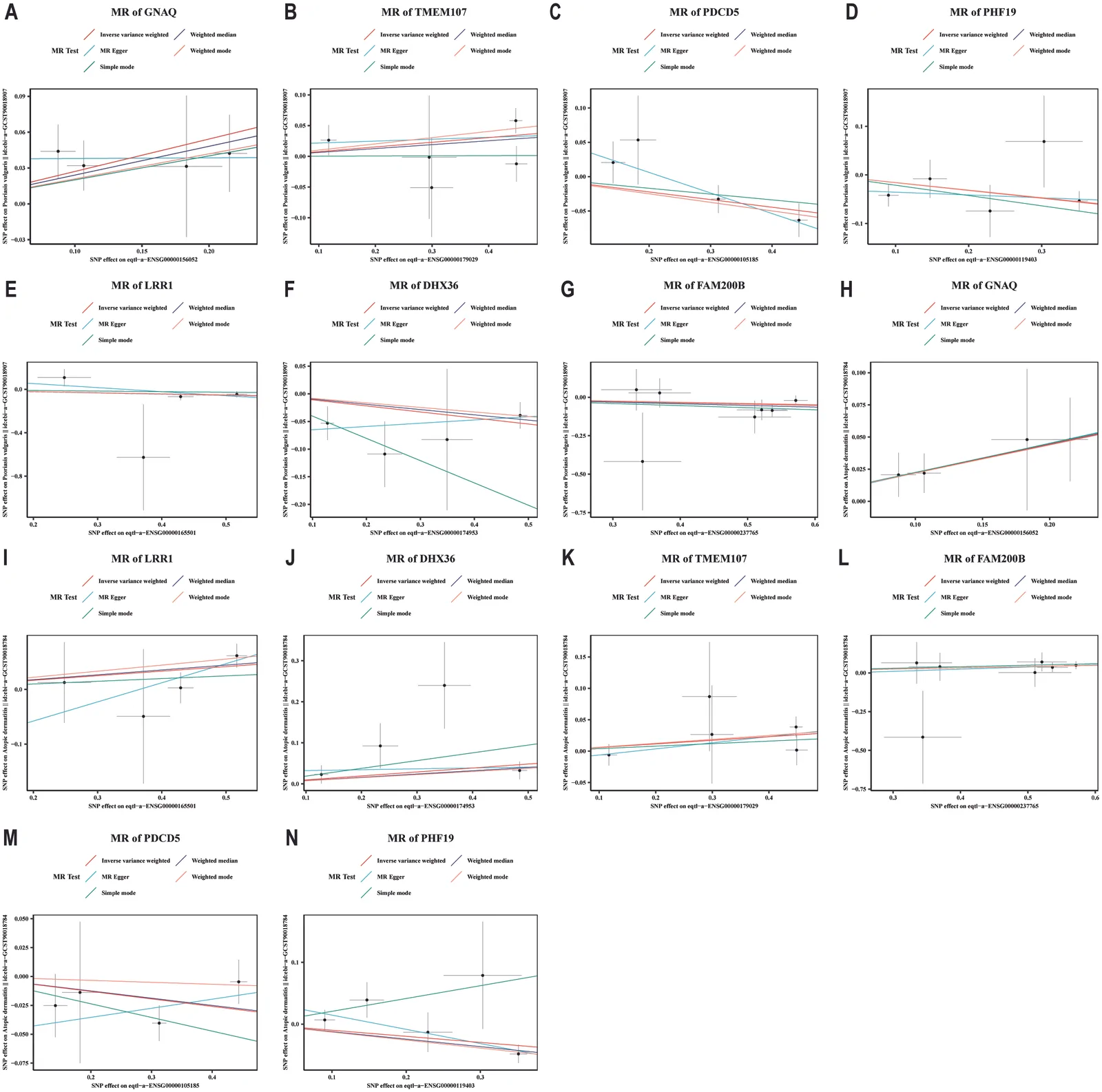
MR identifies shared causal genes for PV and AD. **(A–G)** Scatter plots showing MR results for seven candidate genes in PV: GNAQ **(A)**, TMEM107 **(B)**, PDCD5 **(C)**, PHF19 **(D)**, LRR1 **(E)**, DHX36 **(F)**, and FAM200B **(G)**, with effect direction indicated (positive/risk or negative/protective). **(H–N)** Scatter plots showing MR results for the same seven candidate genes in AD: GNAQ **(H)**, LRR1 **(I)**, DHX36 **(J)**, TMEM107 **(K)**, FAM200B **(L)**, PDCD5 **(M)**, and PHF19 **(N)**, with effect direction indicated.

To further evaluate the causal relevance of these candidate genes, MR analysis was performed for both PV and AD. Using the IVW method, significant associations were identified between candidate genes and both diseases. Specifically, 59 PV-related genes were found to exhibit p-values below 0.05 (**Supplementary Table 7**). For PV, 36 genes (such as PDCD5, PHF19, LRR1, DHX36, and FAM200B) were identified as protective factors against PV (odds ratio (OR)< 1). Conversely, 23 genes, including GNAQ and TMEM107, were acknowledged as risk factors (OR > 1). By applying the IVW method, scatter plots demonstrated positive slopes for 23 genes, including GNAQ and TMEM107, indicating their potential role as risk factors for PV (**Figures 5A, B**; **Additional file 1**). In contrast, the remaining 36 genes (such as PDCD5, PHF19, LRR1, DHX36, and FAM200B) displayed negative slopes, suggesting their possible function as protective factors (**Figures 5C–G**; **Additional file 1**). The findings from the forest plots aligned with those from the scatter plots (**Additional file 2**). Furthermore, funnel plots illustrated a symmetrical and uniform distribution of SNPs across all 59 genes, indicating compliance with Mendel’s second law (**Additional file 3**). Moreover, among these genes, 57 genes (e.g., GNAQ and TMEM107) showed a p-value greater than 0.05 without heterogeneity, leading to the application of the fixed-effects model. The other two genes (HLA-DRB5 and BTN3A2), which had a p-value less than 0.05 and demonstrated heterogeneity, were analyzed using the random-effects model (**Supplementary Table 8**). Besides, except for MTR which showed a p-value< 0.05 in the horizontal pleiotropy test, the remaining 58 genes each demonstrated p-values > 0.05, suggesting no evidence of horizontal pleiotropy and indicating reliable results (**Supplementary Table 9**). The LOO analysis conducted on 58 genes demonstrated that the exclusion of any single SNP had only a minimal effect on PV risk (**Additional file 4**). The Steiger test was utilized to validate the correct orientation of 58 genes, thereby revealing a unidirectional causal relationship with PV. The genes exhibiting a confirmed causal direction (Steiger-direction = TRUE and p-value< 0.05) were classified as PV-feature genes (**Supplementary Table 10**). Based on the IVW results for AD, 66 candidate genes were found to exhibit statistically significant associations with AD (p-value< 0.05). Among these, 32 genes (e.g., GNAQ, LRR1, DHX36, TMEM107, and FAM200B) were identified as risk factors (OR > 1), whereas 34 genes were recognized as protective factors (OR< 1), including PDCD5 and PHF19 (**Figures 5H–N**; **Supplementary Table 11**). Detailed data concerning the relevant scatter plots, forest plots, and funnel plots are presented in **Additional files 5-7**. Subsequently, validation via sensitivity analysis confirmed that 66 genes demonstrated a unidirectional causal relationship with AD and were consequently classified as AD-feature genes. The pertinent findings are outlined in **Supplementary Table 12** and **Additional file 8**.

### Functional annotation of MR-validated shared genes links Treg dysfunction to epigenetic remodeling and proteostasis

Having established causal relationships for 59 PV-feature and 66 AD-feature genes through MR, we identified the intersection of these two gene sets, which yielded seven shared causal genes: PDCD5, PHF19, GNAQ, LRR1, DHX36, TMEM107, and FAM200B (**Supplementary Figure 5A**). To elucidate the biological processes underlying their shared causal relevance, we performed GO and KEGG enrichment analyses. GO analysis revealed significant enrichment in stem cell differentiation, dendritic cell cytokine regulation, and mRNA processing, alongside G-quadruplex DNA binding and telomerase RNA binding (**Supplementary Figure 5B**; **Supplementary Table 13**). KEGG pathway analysis further identified Polycomb repressive complex signaling, cortisol synthesis, and calcium reabsorption (**Supplementary Figure 5C**; **Supplementary Table 14**). These convergent annotations position the seven shared genes at the intersection of three core regulatory axes: epigenetic control, protein homeostasis, and immune-endocrine crosstalk, providing a mechanistic basis for their causal relevance to Treg dysfunction in both PV and AD.

PPI network analysis further revealed a direct protein-protein interaction between PHF19 and LRR1, while the remaining five genes formed discrete nodes without detectable physical interactions (**Supplementary Figure 5D**). The PHF19-LRR1 interaction is particularly notable given that PHF19 functions as a core accessory subunit of the Polycomb repressive complex 2 (PRC2), responsible for H3K36me2 recognition and PRC2 recruitment to chromatin targets to establish H3K27me3-mediated transcriptional silencing. As a protective factor in both PV and AD, PHF19 presumably exerts its disease-suppressive effect through PRC2-dependent epigenetic regulation. The physical coupling of PHF19 with LRR1, a leucine-rich repeat protein implicated in cell cycle checkpoint control, raises the possibility that PHF19-mediated epigenetic repression and cell cycle regulation are functionally coordinated, and that disruption of this coordination may contribute to the shared Treg dysfunction observed in both diseases. Beyond this pairwise protein interaction, gene set enrichment analysis of all seven shared genes revealed collective enrichment in the proteasome and oxidative phosphorylation pathways (**Supplementary Figure 5E**), reinforcing the interpretation that disruption of protein turnover and energy metabolism constitutes a shared pathogenic axis in PV and AD.

### Expression characteristics, molecular regulatory networks, and drug/compound interactions of co-expressed genes in PV and AD

**Figure 6:**
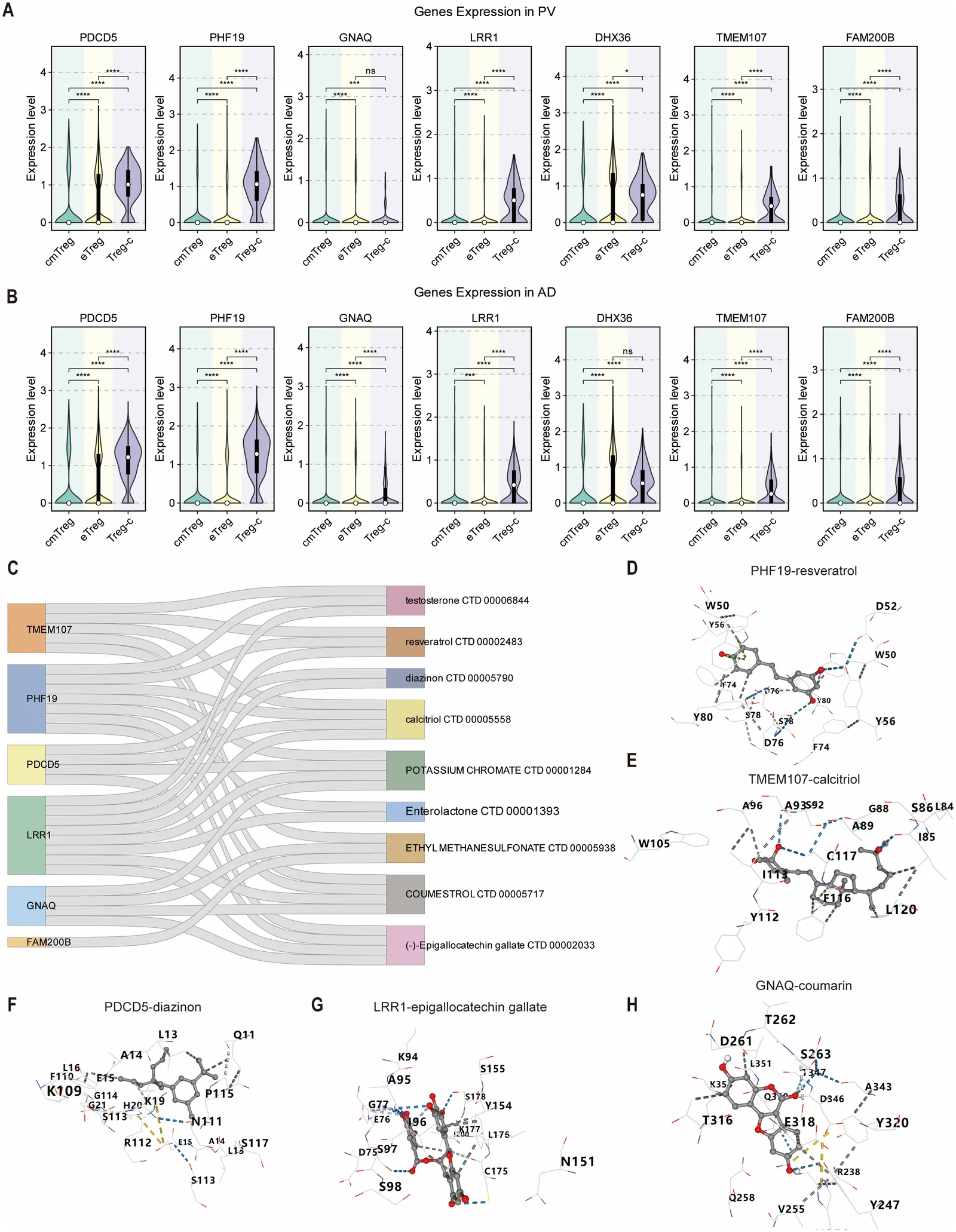
Expression patterns of co-expressed hub genes across Treg subsets and their drug/compound interaction profiles. **(A)** Violin plots showing expression of seven shared genes (PDCD5, PHF19, GNAQ, LRR1, DHX36, TMEM107, and FAM200B) across cmTreg, eTreg, and Treg-c subclusters between HC and PV samples, with statistical significance indicated. **(B)** Violin plots showing expression of the same seven shared genes across cmTreg, eTreg, and Treg-c subclusters between HC and AD samples, with statistical significance indicated. **(C)** Sankey diagram illustrating drug-gene prediction associations between the seven shared genes and their predicted targeting compounds. **(D)** Three-dimensional molecular docking structure of PHF19 with resveratrol. **(E)** Three-dimensional molecular docking structure of TMEM107 with calcitriol. **(F)** Three-dimensional molecular docking structure of PDCD5 with diazinon. **(G)** Three-dimensional molecular docking structure of LRR1 with epigallocatechin gallate. **(H)** Three-dimensional molecular docking structure of GNAQ with coumarin. Data are presented as mean ± SEM. Statistical significance was determined by unpaired Student’s t-test (*P < 0.05, ***P < 0.001, ****P < 0.0001, *ns*, not significant).

Having functionally annotated the seven MR-validated shared genes at the pathway level, we next examined their expression patterns across the three Treg subsets to determine whether their differential distribution aligns with the Treg subset remodeling. In PV, PDCD5, PHF19, LRR1, TMEM107, and FAM200B showed highly significant expression differences across cmTreg, eTreg, and Treg-c, while DHX36 showed significant differences and GNAQ showed no difference between eTreg and Treg-c but significant differences between cmTreg and the other two subsets (**Figure 6A**; **Supplementary Table 15**). In AD, the same general pattern was observed: DHX36 showed no difference between eTreg and Treg-c, whereas the remaining six genes displayed highly significant differences across all three subsets (**Figure 6B**; **Supplementary Table 15**). The consistent discrimination of these seven genes between cmTreg and the two disease-expanded subsets (eTreg and Treg-c) positions them as molecular correlates of the Treg subset remodeling that characterizes both PV and AD.

To identify the upstream regulatory drivers of this shared expression signature, we first constructed a transcription factor (TF) regulatory network. TF prediction identified ATF2 and MYBL2 as two master regulators shared by six of the seven genes (all except PDCD5 for ATF2; all except GNAQ for MYBL2) (**Supplementary Figure 6A**; **Supplementary Table 16**). ATF2 responds to stress and inflammatory signals, while MYBL2 regulates cell cycle progression; their convergent control over the majority of the shared gene set suggests that these genes are coordinately activated by inflammatory stress and proliferative cues in diseased Treg subsets. Subsequently, lncRNA targets of these intersecting miRNAs were predicted using the StarBase database, resulting in the identification of 48 relevant lncRNAs (**Supplementary Figure 6B**; **Supplementary Table 16**). The constructed lncRNA–miRNA–mRNA regulatory network visually illustrated the intricate interactions among non-coding RNAs and mRNAs, indicating multi-layered transcriptional and post-transcriptional control. Finally, we explored the therapeutic translational potential of these findings through drug prediction and molecular docking. Nine candidate compounds were predicted to target six of the seven genes (**Figure 6C**; **Supplementary Table 17**). Molecular docking predicted strong binding affinities (binding free energy< -5 kcal/mol) for five of six receptor-ligand pairs tested. The PHF19-resveratrol complex showed the most favorable binding energy (-8.6 kcal/mol), followed by GNAQ-coumarin (-8.4 kcal/mol), LRR1-epigallocatechin gallate (-7.7 kcal/mol), TMEM107-calcitriol (-6.3 kcal/mol) and PDCD5-diazinon (-5.8 kcal/mol); only the FAM200B-ethyl methanesulfonate complex fell below the threshold (-4.3 kcal/mol) (**Table 3**; **Figures 6D–H**; **Supplementary Figure 6C**). The exceptional affinity of resveratrol for PHF19—combined with PHF19’s protective causal status in both diseases and its established role as a PRC2 accessory subunit—positions the PHF19-resveratrol interaction as the most promising candidate for experimental validation.

**Table 3:** The binding free energy between co-expressed genes and drugs.

Drugs	Gene name	Binding free energy (kcal/mol)
Calcitriol	TMEM107	-6.3
Resveratrol	PHF19	-8.6
Diazinon	PDCD5	-5.8
Epigallocatechin gallate	LRR1	-7.7
Coumarin	GNAQ	-8.4
Ethyl methanesulfonate	FAM200B	-4.3

### Resveratrol ameliorates PV and AD-like skin lesions by restoring Treg subset homeostasis through activation of the PHF19-PRC2 axis

**Figure 7:**
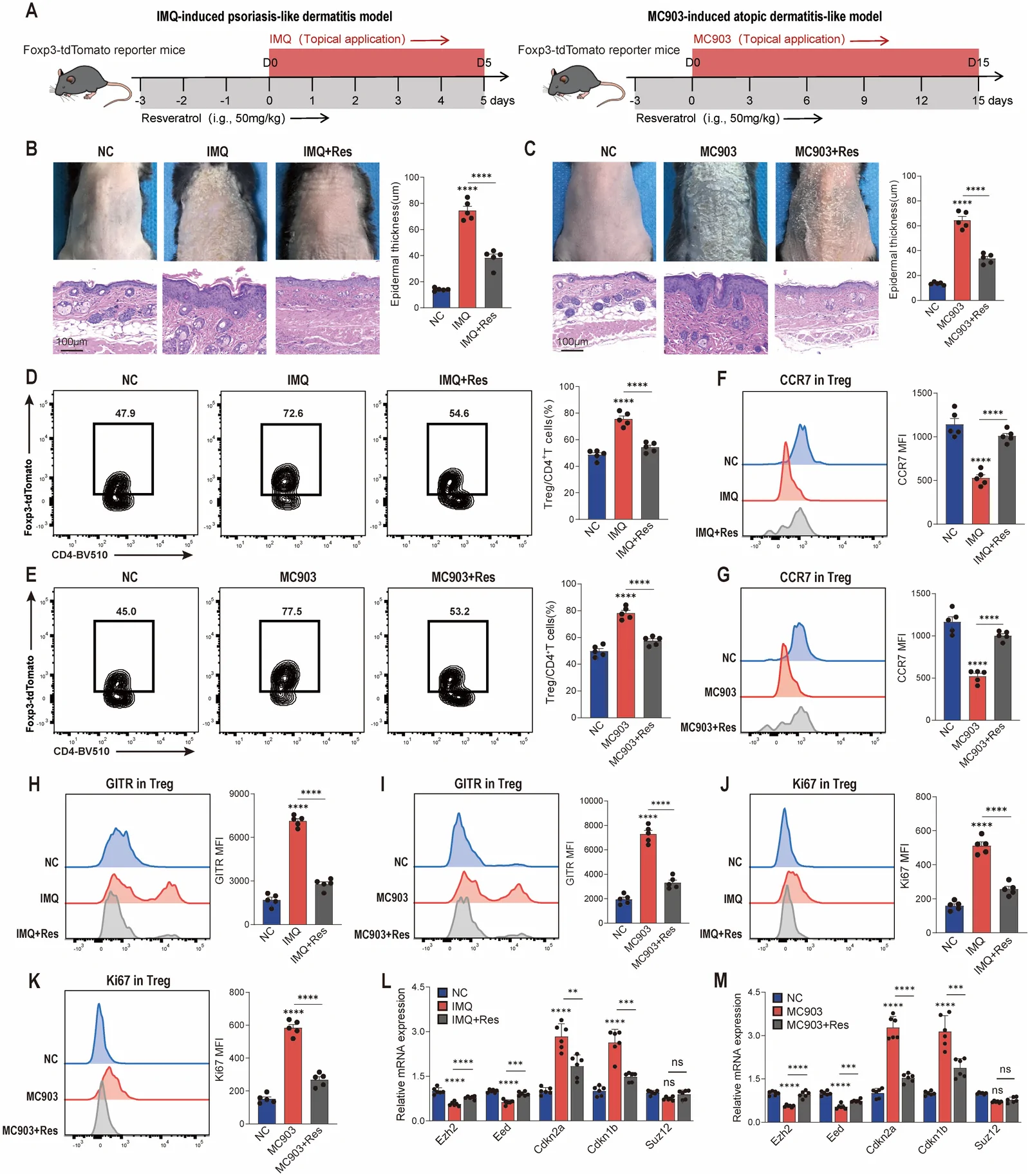
Resveratrol ameliorates skin lesions and restores Treg subset homeostasis via PHF19-PRC2 axis activation. **(A)** Schematic of the IMQ-induced psoriasiform dermatitis model (left, Day 0–5) and MC903-induced AD-like model (right, Day 0–15) in Foxp3-tdTomato reporter mice with resveratrol (Res) treatment (50 mg/kg, intragastric). **(B)** Representative skin photographs and H&E-stained sections (left) and quantification of epidermal thickness (right) for NC, IMQ, and IMQ+Res groups. Scale bar, 100 μm. **(C)** Representative skin photographs and H&E-stained sections (left) and quantification of epidermal thickness (right) for NC, MC903, and MC903+Res groups. Scale bar, 100 μm. **(D)** Flow cytometry plots (left) and quantification (right) of Treg proportions among CD4^+^T cells in the IMQ model. **(E)** Flow cytometry plots (left) and quantification (right) of Treg proportions among CD4^+^T cells in the MC903 model. **(F)** CCR7 MFI in Treg cells with representative histograms (left) and quantification (right) in the IMQ model. **(G)** CCR7 MFI in Treg cells with representative histograms (left) and quantification (right) in the MC903 model. **(H)** GITR MFI in Treg cells with representative histograms (left) and quantification (right) in the IMQ model. **(I)** GITR MFI in Treg cells with representative histograms (left) and quantification (right) in the MC903 model. **(J)** Ki67 MFI in Treg cells with representative histograms (left) and quantification (right) in the IMQ model. **(K)** Ki67 MFI in Treg cells with representative histograms (left) and quantification (right) in the MC903 model. **(L)** Relative mRNA expression of Ezh2, Eed, Cdkn2a, Cdkn1b, and Suz12 in Treg cells from the IMQ model measured by qRT-PCR. **(M)** Relative mRNA expression of Ezh2, Eed, Cdkn2a, Cdkn1b, and Suz12 in Treg cells from the MC903 model measured by qRT-PCR. Data are presented as mean ± SEM. Statistical analysis was performed using unpaired Student’s *t*-test (*P < 0.05, **P < 0.01, ***P < 0.001, ****P < 0.0001, *ns*, not significant).

To validate the PHF19-resveratrol interaction therapeutically, we used the Foxp3-tdTomato reporter mouse models described above and treated IMQ- or MC903-challenged mice with resveratrol or vehicle control (**Figure 7A**). Resveratrol treatment significantly ameliorated skin lesion severity in both models, as evidenced by reduced erythema, scaling, and epidermal thickness on H&E staining (**Figures 7B, C**). To determine whether resveratrol exerts its therapeutic effects through modulation of Treg cells, we analyzed skin-infiltrating Treg populations by flow cytometry. In both the IMQ and MC903 models, the proportion of total Treg cells among CD4^+^ T cells was markedly elevated in vehicle-treated diseased mice compared with controls, while resveratrol treatment significantly reduced these elevated total Treg proportions (**Figures 7D, E**), indicating suppression of pathological Treg expansion.

We next asked whether resveratrol specifically corrects the abnormal Treg subset remodeling observed in both disease models. In vehicle-treated mice, the mean fluorescence intensity (MFI) of CCR7—a cmTreg-associated marker—in Treg cells was significantly decreased, whereas the MFI of GITR and Ki67—markers associated with eTreg and Treg-c, respectively—was markedly increased in both models, confirming the Treg triad shift at the protein level. Resveratrol reversed these alterations: CCR7 MFI was significantly restored, and GITR and Ki67 MFI were correspondingly decreased (**Figures 7F–K**). These results demonstrate that resveratrol reverses the cmTreg-depleted, eTreg/Treg-c-expanded pattern in both disease models.

We next examined whether resveratrol modulates the PHF19-PRC2 axis to produce these Treg-restorative effects. Molecular docking predicted direct binding of resveratrol to the PHF19 protein; if this binding affects PHF19 function, it should be reflected in downstream PRC2 activity. We sorted Treg cells from mice and performed qRT-PCR. Analysis of PRC2 core components revealed that EZH2 and EED mRNA levels were significantly decreased in Treg cells from both disease models, suggesting impaired PRC2 complex integrity under inflammatory conditions. Resveratrol treatment reversed these reductions, consistent with PHF19-dependent restoration of PRC2 assembly and catalytic activity. Conversely, the established PRC2-repressed target genes CDKN2A (p16) and CDKN1B (p27) showed markedly elevated mRNA levels in both model groups, reflecting loss of H3K27me3-mediated transcriptional silencing when PRC2 function is compromised. Resveratrol treatment significantly reduced both CDKN2A and CDKN1B mRNA expression, demonstrating that resveratrol-mediated PHF19 activation reinstates PRC2-dependent epigenetic repression at these cell cycle regulatory loci. SUZ12 showed a similar but non-significant trend. Importantly, this regulatory pattern was consistent across both the IMQ and MC903 models (**Figures 7L, M**). These results collectively indicate that resveratrol restores Treg cell abnormalities in PV and AD potentially through functional engagement of the PHF19-PRC2 axis.

Collectively, these results establish a coherent mechanistic chain: resveratrol binds PHF19, is predicted to functionally activate PRC2 assembly and H3K27me3 deposition, re-silences p16 and p27, normalizes Treg subset differentiation—specifically, recovery of cmTreg proportions and reduction of eTreg and Treg-c expansion—and thereby attenuates cutaneous inflammation in both disease models. These findings validate PHF19 as a druggable target and support resveratrol as a PHF19 activator for therapeutic restoration of Treg functional balance in PV and AD.

## Discussion

The present study integrates scRNA-seq, MR, and experimental pharmacology to demonstrate that PV and AD share a common Treg-centered pathogenic architecture. Our findings position the triad of cmTreg depletion, Treg-c expansion, and eTreg accumulation as a shared pathogenic hallmark, underpinned by aberrant differentiation trajectories, MIF-mediated communication remodeling, and a seven-gene molecular signature with PHF19 at its epigenetic core. Experimental validation further establishes that resveratrol activates the PHF19-PRC2 axis to restore Treg subset homeostasis, providing a basis for targeted therapeutic intervention.

The heterogeneity of Treg subsets in human skin has been characterized by single-cell transcriptomic studies, suggesting that these subsets should be interpreted as transcriptional states along tissue-adaptive Treg programs rather than rigid lineage endpoints (26). Following this established classification framework, we resolved three transcriptionally distinct Treg subsets in lesional skin: cmTreg (CCR7^+^CD62L^+^), which maintains systemic immune surveillance through lymphoid tissue recirculation; eTreg (GITR^+^PRDM1^+^), which executes tissue-adapted effector-phase immunosuppression; and Treg-c (MKI67^+^TOP2A^+^), which serves as the proliferative engine continuously replenishing the regulatory pool. Under healthy conditions, these three subsets maintain a hierarchical equilibrium: cmTreg provides a stable long-lived reservoir for self-renewal and immune tolerance, Treg-c generates new regulatory cells through active proliferation, and eTreg delivers potent suppressive function at inflamed sites. In both PV and AD, this organized architecture undergoes characteristic disruption. cmTreg is nearly absent (828 to 5 cells in PV; 784 to 2 in AD), signaling collapse of the long-lived regulatory reservoir that normally sustains immune tolerance through lymphoid tissue-based renewal. Treg-c expands dramatically (7 to 215 in PV; 7 to 375 in AD), likely reflecting a compensatory proliferative response by the residual regulatory compartment; however, the quantitative mismatch between cmTreg loss (782–823 cells) and Treg-c gain (208–368 cells) indicates this compensation is insufficient to restore the original architecture. Notably, eTreg also accumulates (547 to 3578 in PV; 542 to 4911 in AD), yet this numerical increase does not imply enhanced suppressive capacity. Our pseudotime analysis reveals that these expanded eTregs fail to complete terminal differentiation and instead distribute broadly across immature stages, suggesting a functionally incompetent phenotype that cannot effectively maintain immune homeostasis despite their increased numbers. This triad thus represents a qualitative rather than merely quantitative defect in Treg-mediated immune regulation.

The independent validation of this remodeling signature in Foxp3-tdTomato reporter mice subjected to IMQ-induced psoriasis-like and MC903-induced AD-like dermatitis confirms that the Treg triad is an evolutionarily conserved response to sustained cutaneous inflammation. This conservation positions Treg subset imbalance as a core pathogenic mechanism of chronic inflammatory dermatoses. We propose that this triad reflects a fundamental breakdown of Treg hierarchical organization: the disappearance of cmTreg eliminates the primary reservoir for regulatory renewal, forcing compensatory but misdirected proliferation of Treg-c that feeds functionally immature eTregs into chronically inflamed skin. This model suggests that therapeutic strategies restoring normal Treg subset architecture may have broad applicability across the PV-AD disease spectrum.

Pseudotemporal analysis revealed a fundamental defect in Treg differentiation coordination: cmTreg and Treg-c compartments are prematurely shifted toward advanced pseudotime stages, while the eTreg population appears not to complete terminal maturation. As a result, activated precursors fail to generate functional effectors, perpetuating regulatory insufficiency. Functional enrichment analyses further illuminate the divergent fates of these subsets in disease. eTreg exhibits enhanced oxidative phosphorylation (OXPHOS), reflecting compensatory metabolic reprogramming to meet energy demands in inflamed microenvironments; however, sustained OXPHOS reliance renders these cells vulnerable to reactive oxygen species-mediated damage and functional exhaustion. Conversely, Treg-c shows exclusively downregulated gene enrichment, indicating broad functional suppression. Notably, Treg-c downregulation followed disease-specific patterns: epidermal differentiation and cadherin adhesion pathways were suppressed in PV, whereas carbohydrate transport and LPS response pathways were affected in AD. These context-dependent alterations reflect distinct functional modulation of the Treg-c compartment in each disease setting, implying that these pathway differences may account for divergent clinical presentations.

The intercellular communication landscape underwent equally striking reorganization. In healthy skin, cmTreg served as the dominant signaling hub; in disease, network control shifted to a dual-center architecture jointly governed by Treg-c and eTreg. MIF-(CD74+CXCR4/CD44) signaling emerged as a conserved axis of enhancement in both diseases, with cmTreg withdrawing from signal transmission, Treg-c emerging as the primary sender, and eTreg becoming the predominant receiver. Because MIF signaling modulates Treg functional capacity and inflammatory amplification (58), this network rewiring suggests that loss of cmTreg hub function disrupts tolerogenic communication patterns and enables inflammatory signal propagation through alternative cellular routes. The conservation of this MIF circuit remodeling across both PV and AD positions pharmacological interruption of MIF-(CD74+CXCR4/CD44) signaling as a potential disease-agnostic strategy.

To identify molecular drivers of this conserved Treg disruption, we integrated differential expression analysis with MR and identified seven shared causal genes: PDCD5, PHF19, GNAQ, LRR1, DHX36, TMEM107, and FAM200B. Functional enrichment highlighted PRC2-mediated epigenetic signaling, proteasomal protein homeostasis, and immune-endocrine cross-talk as convergent biological axes. Protein-protein interaction mapping revealed a direct physical interaction between PHF19 and LRR1, positioning PHF19 at the center of the shared molecular network. As a core accessory subunit of PRC2, PHF19 recognizes H3K36me2 marks and recruits the complex to chromatin targets to establish H3K27me3-mediated transcriptional silencing (59). Its protective status in both diseases raises the possibility that reduced PHF19 expression might compromise PRC2 targeting efficiency, derepresses cell-cycle inhibitory genes, and destabilizes the Treg differentiation program.

Molecular docking predicted resveratrol to be the compound with the strongest PHF19 binding affinity. *In vivo* validation demonstrated that resveratrol treatment significantly improved skin lesions in both IMQ psoriasiform and MC903 atopic dermatitis models while reversing the Treg triad—restoring cmTreg proportions and attenuating eTreg and Treg-c expansion. Mechanistically, resveratrol rescued EZH2 and EED mRNA expression, consistent with restored PRC2 complex integrity, and concurrently reduced expression of CDKN2A (p16) and CDKN1B (p27), two established PRC2-repressed targets that negatively regulate cell cycle progression and Treg differentiation. These results establish a coherent mechanistic chain: resveratrol binds and activates PHF19, restores PRC2 assembly and H3K27me3 deposition, re-silences p16/p27, normalizes Treg differentiation hierarchy, attenuates inflammation, and produces measurable disease improvement. This tolerance-restoring paradigm may differ fundamentally from cytokine-suppressing biologics that do not correct underlying Treg dysfunction, offering a potential approach to the immune drift phenomenon.

Several limitations should be acknowledged. First, while we propose that Treg subset remodeling constitutes a plausible cellular mechanism underlying immune drift, we acknowledge that direct experimental evidence—such as analysis of biologic-treated patient samples or *in vitro* Treg differentiation assays under Th1/Th17 versus Th2 polarizing conditions—was not available within the current study timeframe. Future prospective studies collecting longitudinal samples from patients undergoing biologic therapy are warranted to directly test this hypothesis. Second, scRNA-seq cohorts were modest, potentially limiting detection of rare subpopulations; future validation using larger independent cohorts is therefore required. Third, MR relies on assumptions that cannot be guaranteed with absolute certainty, and lifelong genetic predispositions may not directly translate to pharmacological intervention. In addition, the precise biochemical mechanism by which resveratrol modulates PHF19 activity warrants further structural and functional investigation.

In summary, this study identifies a conserved disruption of Treg subset architecture spanning PV and AD, traces its molecular basis to shared epigenetic dysregulation centered on PHF19-PRC2 signaling, and demonstrates that pharmacological restoration of this axis reverses both the cellular defect and disease phenotype. The resveratrol-PHF19 binding interaction reactivates PRC2 assembly, re-establishes H3K27me3-mediated silencing of p16 and p27, restores Treg differentiation hierarchy, and thereby reduces cutaneous inflammation and improves lesion severity. Future directions include resolving the resveratrol-PHF19 binding interface at the structural level, developing PHF19-specific small-molecule activators with improved bioavailability, and preclinical evaluation of their efficacy in chronic inflammatory skin disease models.

## Data Availability

The datasets presented in this study can be found in online repositories. The names of the repository/repositories and accession number(s) can be found in the article/**Supplementary Material**.

## Glossary

AD: atopic dermatitis
BEAM: Branched Expression Analysis Modeling
cmTreg: central memory regulatory T cell
CTL: cytotoxic T lymphocyte
CTLac: activated cytotoxic T lymphocyte
CTLex: exhausted cytotoxic T lymphocyte
DMSO: dimethyl sulfoxide
EGA: European Genome-Phenome Archive
eTreg: effector regulatory T cell
FDR: false discovery rate
GSEA: gene set enrichment analysis
GWAS: genome-wide association study
HC: healthy control
H&E: hematoxylin and eosin
IMQ: imiquimod
ILC: innate lymphoid cell
IV: instrumental variable
IVW: inverse variance weighted
LOO: leave-one-out
MFI: mean fluorescence intensity
MIF: macrophage migration inhibitory factor
MR: Mendelian randomization
NK: natural killer
OXPHOS: oxidative phosphorylation
OR: odds ratio
PBS: phosphate-buffered saline
PCA: principal component analysis
PHF19: PHD finger protein 19
PRC2: Polycomb repressive complex 2
PV: psoriasis vulgaris
QC: quality control
qRT-PCR: quantitative real-time PCR
scRNA-seq: single-cell RNA sequencing
SNP: single nucleotide polymorphism
Tcm: central memory T cell
TF: transcription factor
Tmm: migratory memory T cell
Tn: naive T cell
Treg: regulatory T cell
Treg-c: cycling regulatory T cell
Trm: tissue-resident memory T cell
UMAP: Uniform Manifold Approximation and Projection
