# Supplementary material for: Shared Treg dysregulation underlies psoriasis vulgaris and atopic dermatitis through the PHF19-PRC2 epigenetic axis
Source: Front Immunol. 2026 Aug 20;17:1932788. doi: 10.3389/fimmu.2026.1932788 (PMC13538097; doi:10.3389/fimmu.2026.1932788)
Supplement: Supplementary file 1 [file DataSheet1.pdf]

Supplementary materials

Supplementary Figure:

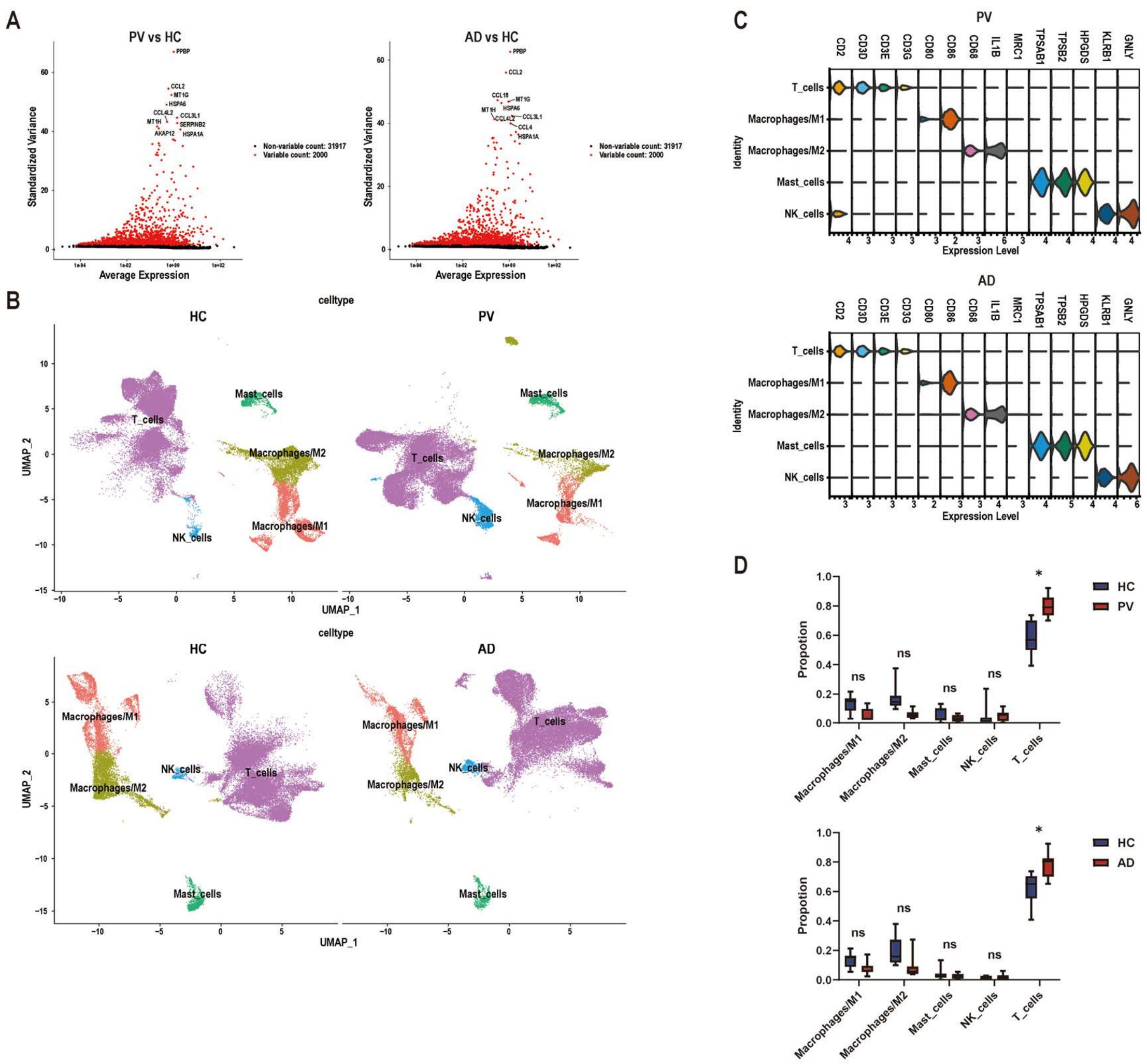

Figure S1. Quality control and clustering of immune cells from PV and AD datasets. (**A**) Scatter plots of highly variable genes (HVGs) showing standardized variance versus average expression for PV vs HC (left) and AD vs HC (right), with HVGs highlighted in red. (**B**) UMAP plots of all immune cells from PV datasets (upper), and AD datasets (lower), annotated as T cells, Macrophages/M1, Macrophages/M2, Mast cells, and NK cells. (**C**) Violin plots showing canonical marker gene expression across cell types in PV datasets (upper) and AD datasets (lower). (**D**) Box plots comparing

immune cell proportions across individual samples in HC vs PV (upper) and HC vs AD (lower), with statistical significance indicated (*$P < 0.05$, ns, not significant).

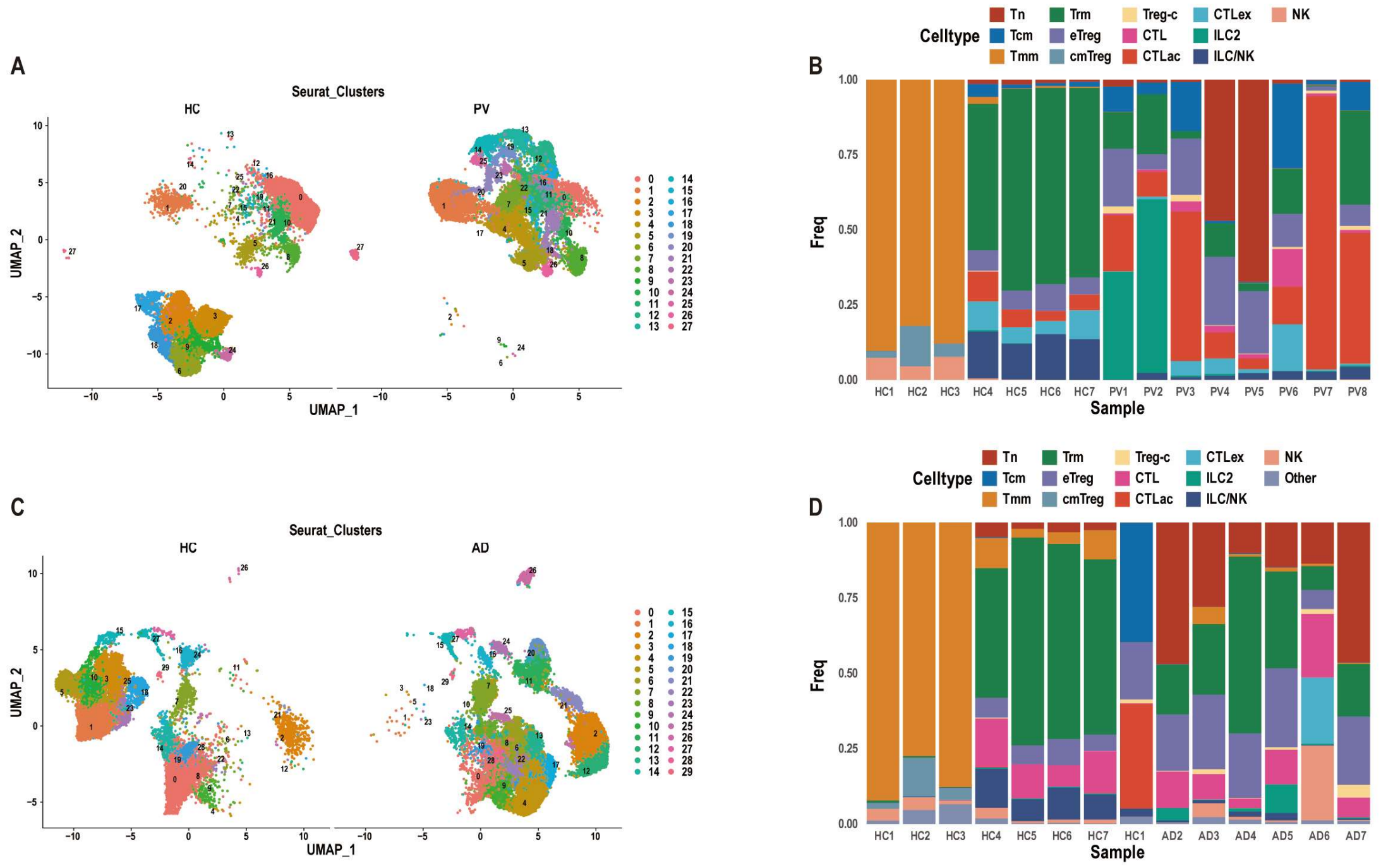

Figure S2. High-resolution T cell clustering reveals Seurat subclusters in PV and AD. (**A**) UMAP plot of 28 Seurat subclusters of T cells from HC and PV samples, colored by cluster identity. (**B**) Stacked bar plots showing the frequency distribution of T cell subpopulation across HC and PV samples. (**C**) UMAP plot of 30 Seurat subclusters of T cells from HC and AD samples, colored by cluster identity. (**D**) Stacked bar plots showing the frequency distribution of T cell subpopulation across HC and AD samples.

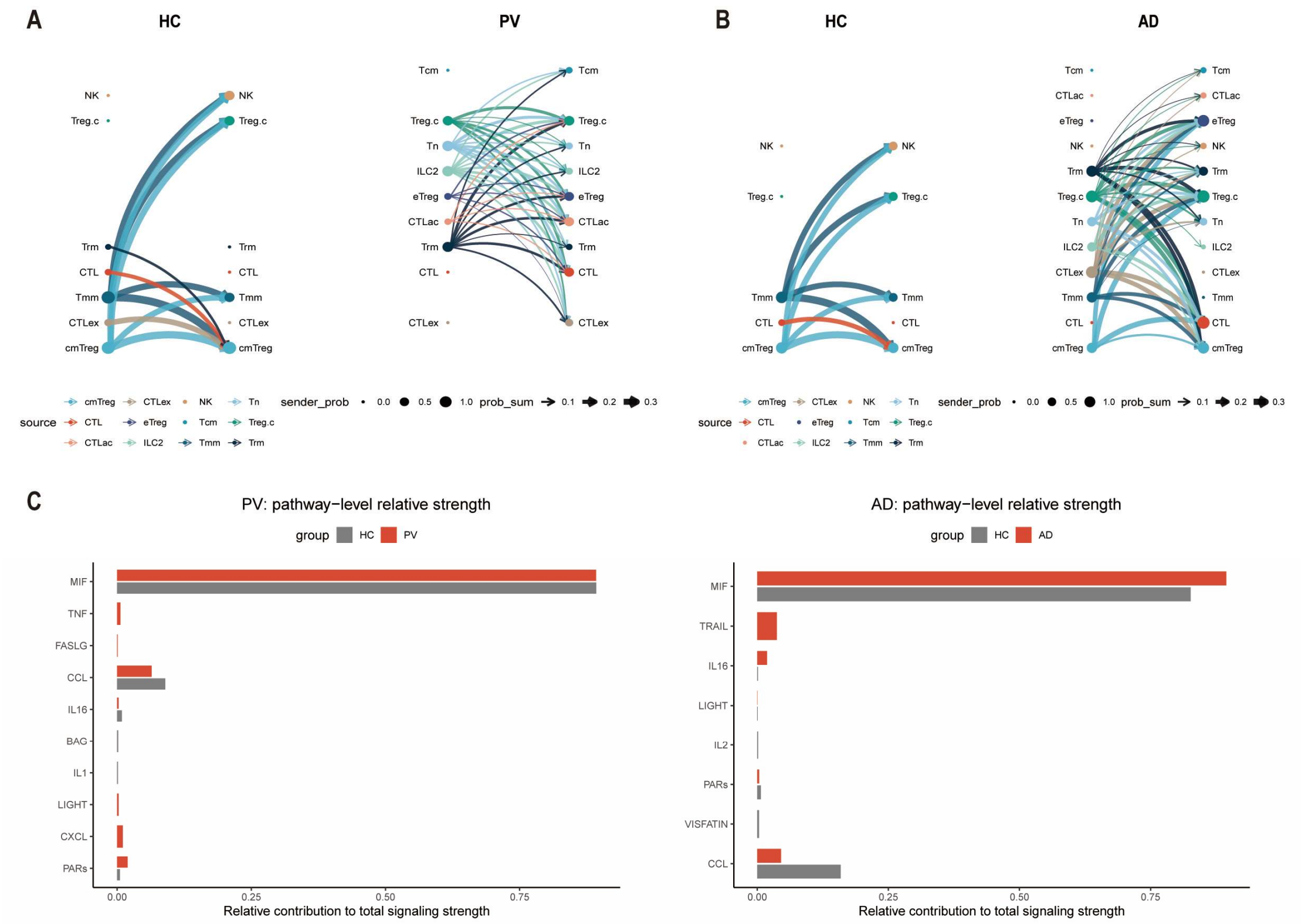

Figure S3. Simplified summary of MIF-centric cell–cell communication networks and pathway-level signaling strength in PV and AD. (**A**) Condensed MIF sender–receiver network for the PV dataset showing redistribution of MIF signal emission from cmTreg (HC, left) to Treg-c/eTreg (PV, right). (**B**) Condensed MIF sender–receiver network for the AD dataset showing analogous MIF emission redistribution from cmTreg (HC, left) to Treg-c/eTreg (AD, right). (**C**) Pathway-level relative contribution to total signaling strength revealing MIF as the dominant shared axis in both diseases, alongside disease-specific pathways (TNF, FASL-FAS, CXCL13-CXCR3 in PV; TNFSF10-TNFRSF10B, IL16-CD4 in AD).

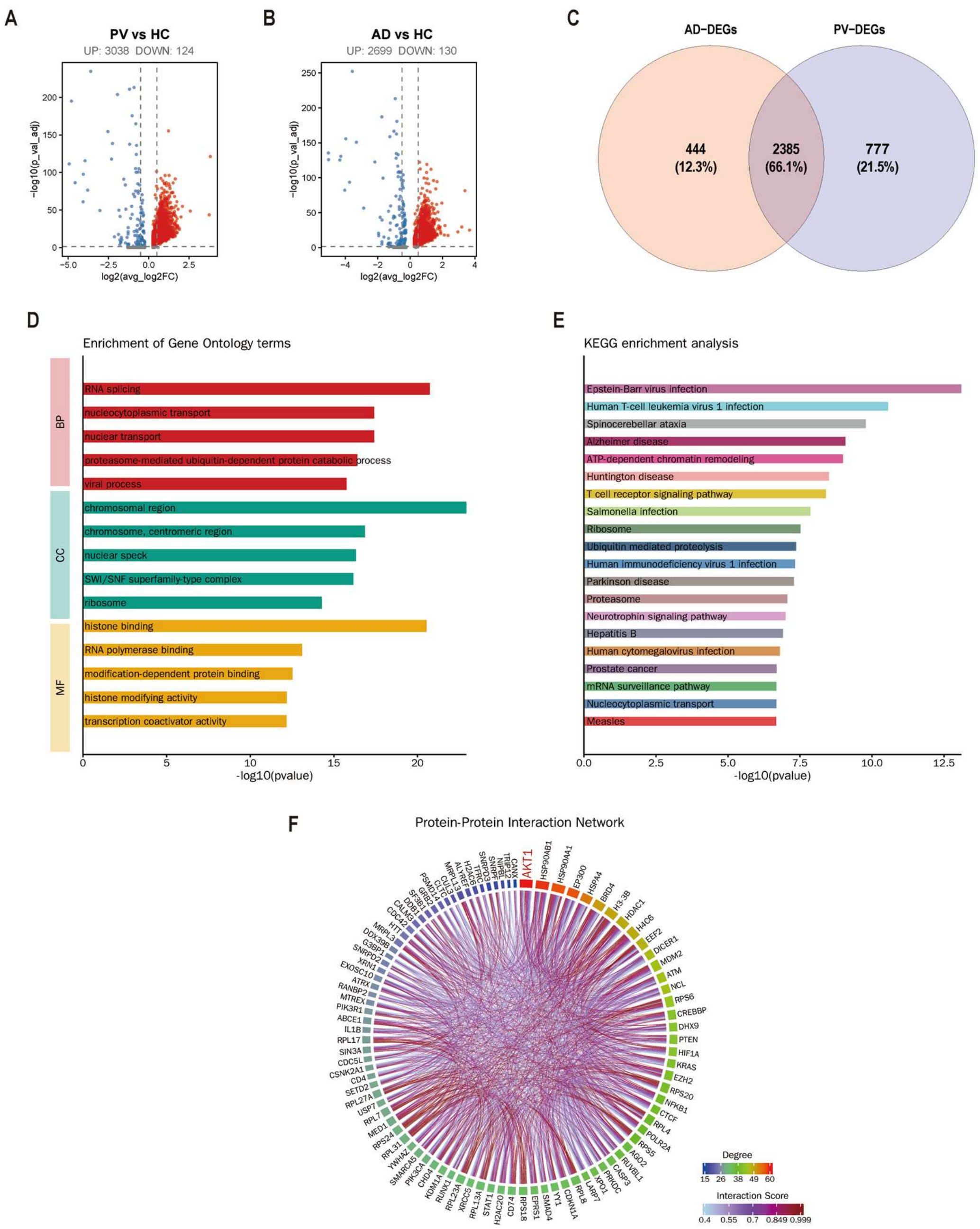

Figure S4. Overlapping DEGs in Treg cells from PV and AD, and corresponding enrichment analysis. (**A**) Volcano plot showing DEGs in Treg cells between PV and HC, with upregulated genes (red) and downregulated genes (blue) highlighted. (**B**) Volcano plot showing DEGs in Treg cells between AD and HC, with upregulated and downregulated genes highlighted. (**C**) Venn diagram showing the overlap of DEGs between PV-DEGs and AD-DEGs, with 2,385 shared genes (66.1% overlap) identified.

(**D**) GO enrichment analysis of overlapping candidate genes, showing biological process (BP), cellular component (CC), and molecular function (MF) categories. (**E**) KEGG pathway enrichment analysis of overlapping candidate genes. (**F**) Protein-protein interaction (PPI) network of the top 2,000 candidate genes, with 93 hub genes retained after removing isolated nodes.

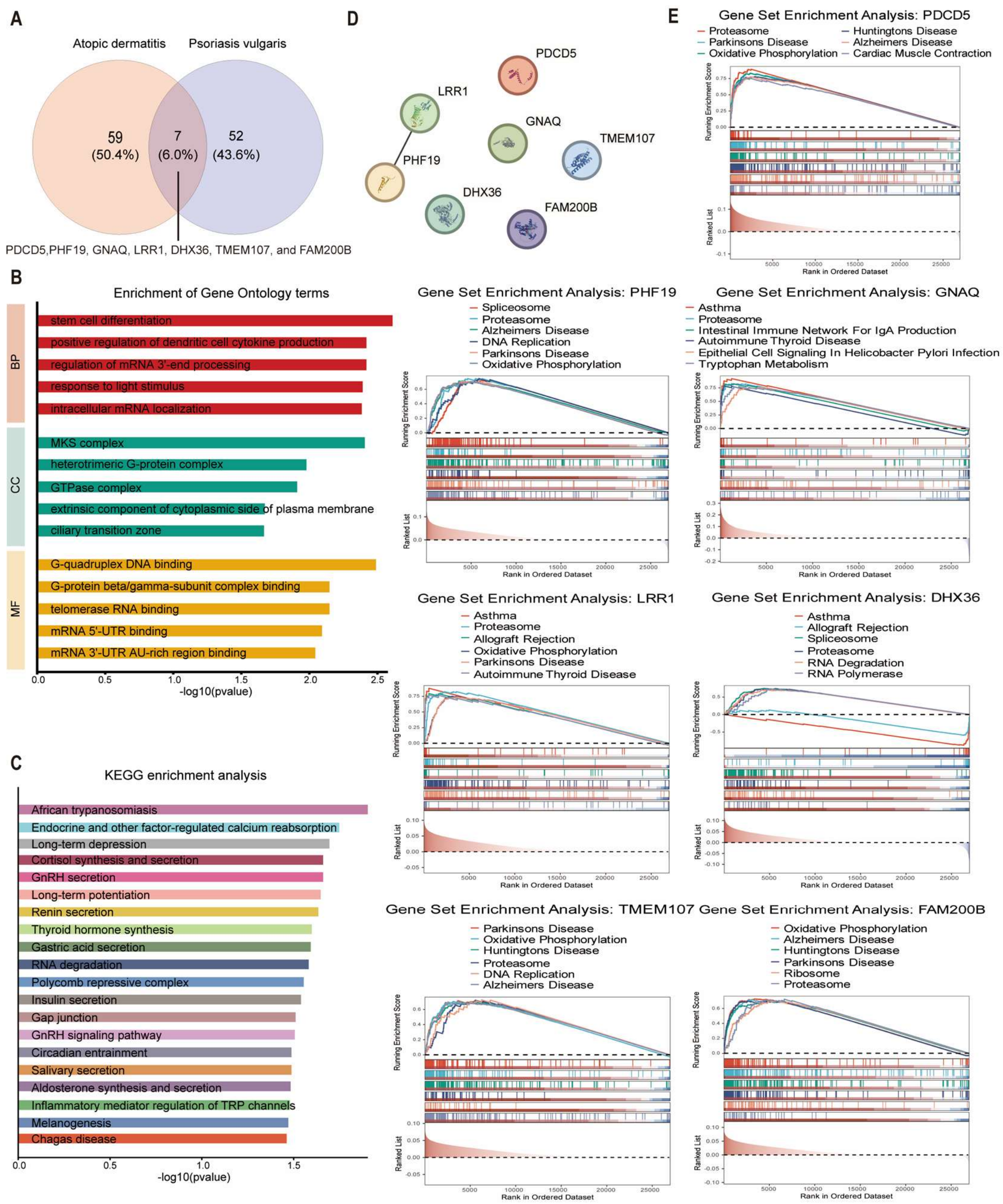

Figure S5. Shared hub genes and their functional enrichment and interaction networks. (**A**) Venn diagram showing the intersection of PV-feature genes and AD-feature genes, identifying 7 shared genes. (**B**) GO enrichment analysis of the 7 shared genes across BP, CC, and MF categories. (**C**) KEGG pathway enrichment analysis of the 7 shared genes. (**D**) PPI network of the 7 shared genes, revealing a direct protein-protein interaction between PHF19 and LRR1. (**E**) Gene set enrichment analysis (GSEA) of

individual shared genes, with separate enrichment plots for each of the 7 genes.

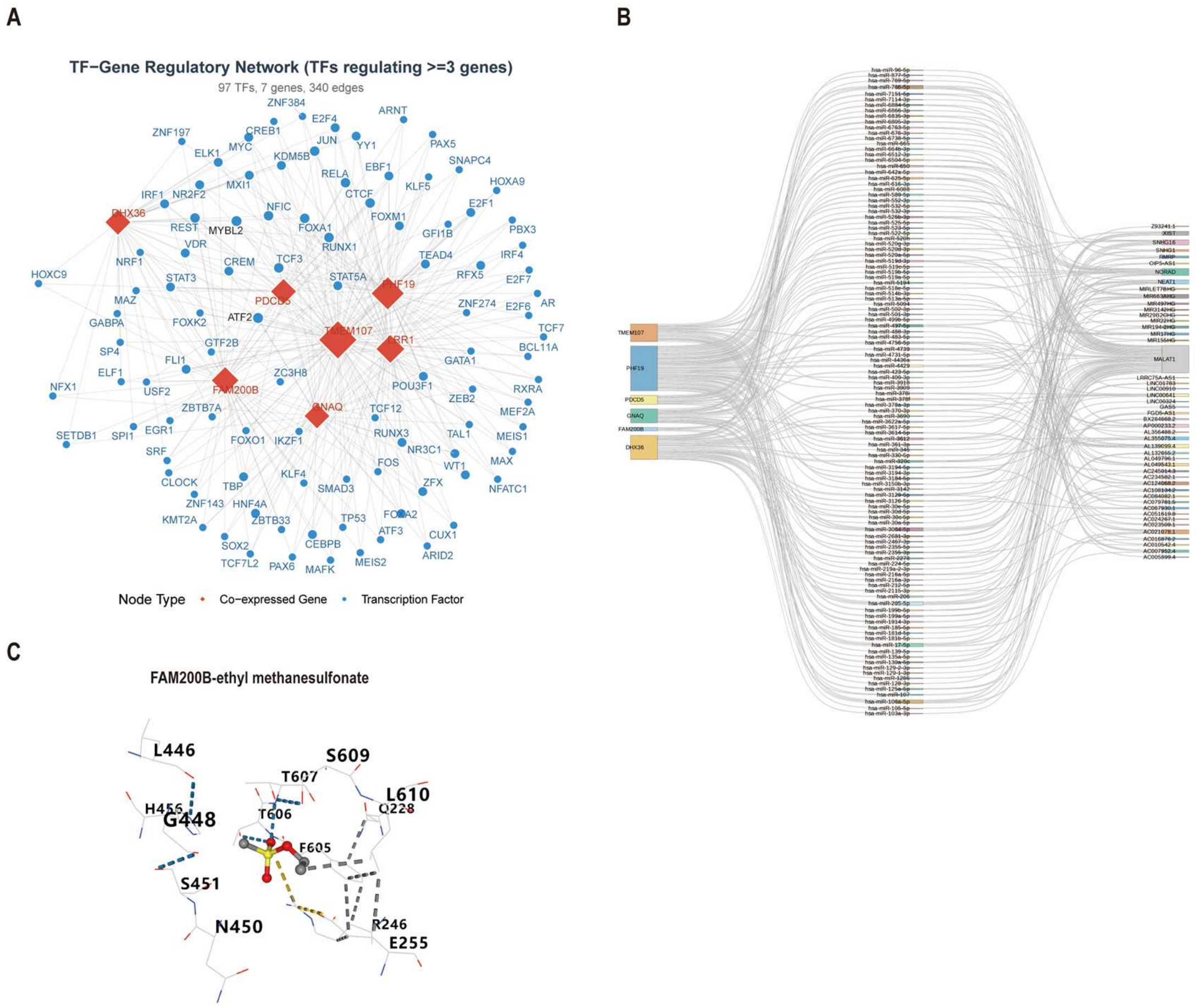

Figure S6. Regulatory networks and molecular docking of shared hub genes. (**A**) Transcription factor (TF) regulatory network of 7 shared genes, with predictions showing the TFs regulating each gene. (**B**) lncRNA–miRNA–mRNA competing endogenous RNA regulatory network for shared genes. (**C**) Three-dimensional molecular docking structure of FAM200B with ethyl methanesulfonate.

Supplementary Tables:

Table S1. GWAS outcome and cis-eQTL exposure datasets summary.

Table S2. F-statistics for all MR-screened genes.

Table S3. Steiger directionality test statistics

Table S4. GO and KEGG enrichment of eTregs in PV patients and AD patients versus healthy controls.

Table S5. GO and KEGG enrichment of Tregs-c in PV patients and AD patients versus healthy controls.

Table S6. GO and KEGG enrichment of 2,358 candidate genes.

Table S7. Candidate genes with potential causal relationship to PV identified via IVW analysis.

Table S8. Heterogeneity test for candidate genes with potential causal relationship to PV.

Table S9. Horizontal pleiotropy test for candidate genes with potential causal relationship to PV.

Table S10. Steiger test for genes passing horizontal pleiotropy test in PV.

Table S11. Candidate genes with potential causal relationship to AD identified via IVW analysis.

Table S12. Sensitivity analysis for candidate genes with potential causal relationship to

AD.

Table S13. GO enrichment analysis of co-expressed genes.

Table S14. KEGG pathway enrichment of co-expressed genes.

Table S15. Differential expression analysis of the seven co-expressed genes (PDCD5, PHF19, GNAQ, LRR1, DHX36, TMEM107, and FAM200B) among Treg subsets (cmTregs, eTregs, and Tregs-c) in PV and AD.

Table S16. Predicted intersecting TF and miRNAs associated with PDCD5, PHF19, GNAQ, LRR1, DHX36, TMEM107, and FAM200B.

Table S17. Drugs/Compounds targeting co-expressed genes.

Additional file:

Additional file 1. Scatter plots of candidate genes Associated with PV (Excluding PDCD5, PHF19, GNAQ, LRR1, DHX36, TMEM107, and FAM200B).

Additional file 2. Forest plots of candidate genes with potential causal relationship to PV.

Additional file 3. Funnel plots of candidate genes with potential causal relationship to PV.

Additional file 4. LOO forest plot for candidate genes with potential causal relationship to PV (based on horizontal pleiotropy test).

Additional file 5. Scatter plots of candidate genes associated with AD.

Additional file 6. Forest plots of candidate genes with potential causal relationship to AD.

Additional file 7. Funnel plots of candidate genes with potential causal relationship to AD.

Additional file 8. LOO forest plot for candidate genes with potential causal relationship to AD (based on horizontal pleiotropy test).
